# Advances in cellulose-based hydrogels: tunable swelling dynamics and their versatile real-time applications

**DOI:** 10.1039/d5ra00521c

**Published:** 2025-04-14

**Authors:** Md. Mahamudul Hasan Rumon

**Affiliations:** a Department of Mathematics and Natural Sciences, Brac University 66 Mohakhali Dhaka 1212 Bangladesh mhrumon.ku@gmail.com

## Abstract

Cellulose-derived hydrogels have emerged as game-changing materials in biomedical research, offering an exceptional combination of water absorption capacity, mechanical resilience, and innate biocompatibility. This review explores the intricate mechanisms that drive their swelling behaviour, unravelling how molecular interactions and network architectures work synergistically to enable efficient water retention and adaptability. Their mechanical properties are explored in depth, with a focus on innovative chemical modifications and cross-linking techniques that enhance strength, elasticity, and functional versatility. The versatility of cellulose-based hydrogels shines in applications such as wound healing, precision drug delivery, and tissue engineering, where their biodegradability, biocompatibility, and adaptability meet the demands of cutting-edge healthcare solutions. By weaving together recent breakthroughs in their development and application, this review highlights their transformative potential to redefine regenerative medicine and other biomedical fields. Ultimately, it emphasizes the urgent need for continued research to unlock the untapped capabilities of these extraordinary biomaterials, paving the way for new frontiers in healthcare innovation.

## Abstract 2

Cellulose-derived hydrogels have emerged as game-changing materials in biomedical research, offering an exceptional combination of water absorption capacity, mechanical resilience, and innate biocompatibility.

## Graphical abstract

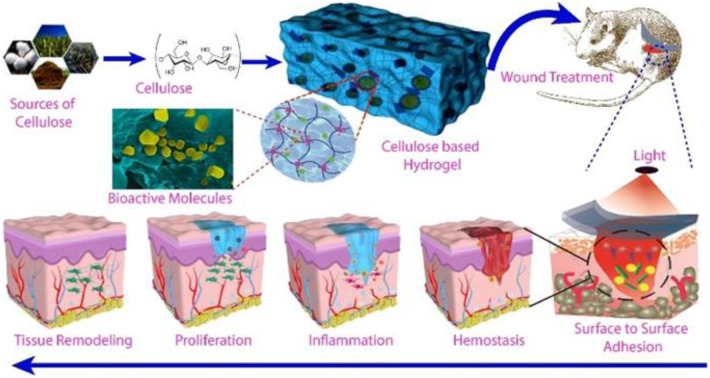

## 1. Introduction

The effectiveness of hydrogels in biomedical applications is fundamentally influenced by their compatibility with natural tissues, mechanical properties, and swelling behaviours.^1,2^ These characteristics are predominantly governed by dynamic bonding interactions, including dynamic covalent bond, hydrogen bonding, hydrophobic interactions, Schiff-base interactions, π–π stacking, ionic bonding, and electrostatic forces.^3–5^ Additionally, external stimuli – such as temperature, pH, salt concentration, light, and electric fields – significantly impact the mechanical and swelling behaviour of gels.^6^ Despite considerable advancements in hydrogel research, conventional materials still encounter limitations, particularly regarding their responsiveness to stimuli like pH-triggered swelling and their other properties.^7,8^ Traditional hydrogels often lack adequate swelling ability and fail to deliver controlled release profiles, which can impede wound healing and other applications.^9^ Such deficiencies may lead to complications, such as adhesion and scab formation at the dressing site, thereby increasing the risk of exogenous infections.^10–12^ Furthermore, their effectiveness in managing infected wounds remains insufficient, highlighting the emergency for the development of bioactive dressings that can promptly address wound infections and promote the overall healing process.^13^

Designing hydrogels for precise bioactive molecule delivery presents significant challenges, particularly in managing antibiotic release.^14^ An excessive release can lead to systemic toxicity, while insufficient release could promote the development of antibiotic-resistant bacteria.^15,16^ As bacterial proliferation occurs, the microenvironment of bio-tissues typically becomes acidic, emphasizing the necessity for hydrogels able to adjust antibiotic release in response to pH variations.^17^ An ideal pH-sensitive hydrogel would degrade in the presence of proliferating bacteria, facilitating the localized release of antibiotics to combat infections. Importantly, this degradation should cease once the wound area returns to a neutral pH.^18^ However, conventional hydrogels often exhibit uncontrolled swelling behaviour, which can compromise their efficacy.^19^ A promising strategy to address this issue involves the incorporation of polymers into hydrogel systems to develop interpenetrating or semi-interpenetrating networks, thus enhancing the mechanical strength of the hydrogels.^20,21^

Cellulose, the most abundant natural polymer, presents substantial potential for hydrogel fabrication due to its inherent hydrophilicity and biodegradability.^22^ Research has successfully demonstrated the development of macroporous hydrogels using cellulose as a support material, with acidic cellulose serving as a pore expander, resulting in hydrogels with high swelling ratios (SR) in aqueous environments.^23,24^ Recent studies have also reported the synthesis of carboxymethyl cellulose (CMC) through the carboxymethylation of cellulose using sodium monochloroacetate in a single solvent system.^25^ These advancements illustrate the growing interest in novel cellulose-based smart hydrogels that exhibit pH and salt sensitivity, underscoring cellulose 's versatility as a candidate for intelligent materials owing to its abundant hydroxyl groups.^26^ Furthermore, using an NaOH/urea aqueous solution as a homogeneous derivation system through environmentally friendly processes has emerged as a favourable method for constructing hydrogel networks *via* both chemical and physical cross-linking.^27^

This comprehensive review aims to provide a thorough overview for the research community, focusing on the mechanistic study of highly swellable gel materials. It will cover fundamental concepts, general classifications, and hydrogel structures, elucidating how the swelling behaviour of cellulose-based hydrogels is influenced by various physicochemical properties and their relevance to biomaterials. A deeper understanding of these mechanisms will enhance knowledge in polymer physics and chemistry, guiding the rational design of custom-tailored cellulose hydrogels for specific applications. From controlled drug delivery systems to responsive materials in smart devices, a nuanced comprehension of swelling behaviour mechanisms will foster innovation. Additionally, the review will highlight recent advancements in integrating highly swellable hydrogel-based materials into biomedical and environmental applications to improve performance and stability. In conclusion, this review will outline existing challenges and potential opportunities, emphasizing the promising future of intelligently designed hydrogels through gelation chemistry in biomedical applications.

## 2. Cellulose derivatives

**Scheme 1:**
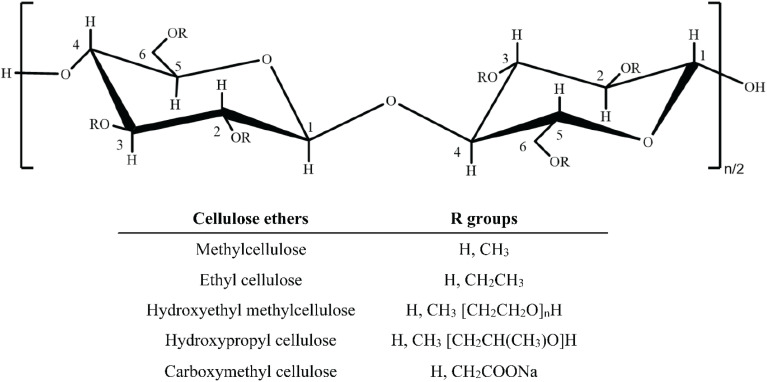
Schematic presentation of cellulose and its various derivatise. The idea of this figure is taken from the ref. 28. Copyright © 2018, Springer Nature.

**Scheme 2:**
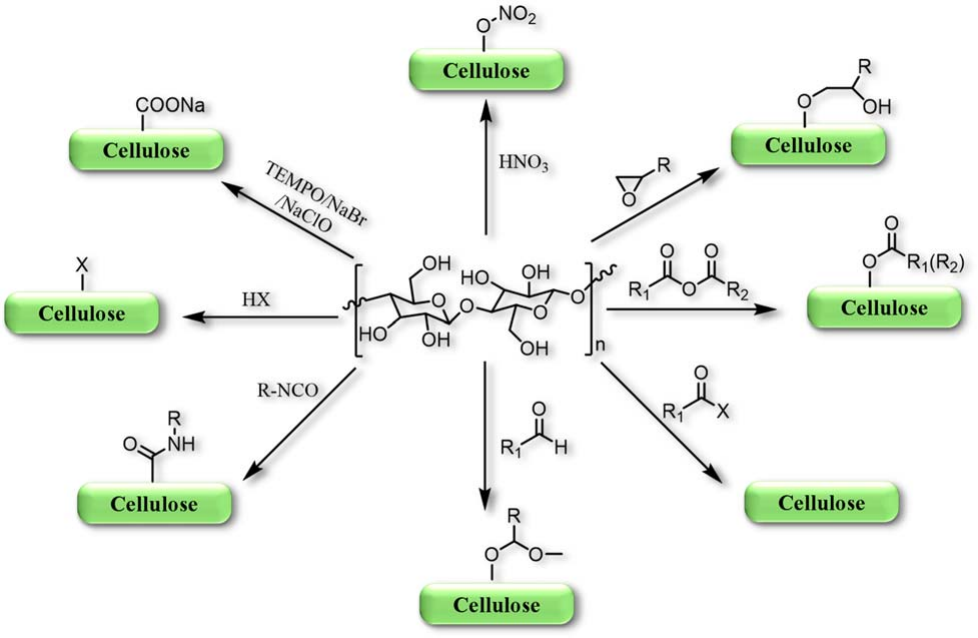
Representation of the various approached for the transformations of cellulose to its derivatives.

Cellulose derivatives can be derived by the etherification process, wherein the OH groups of the glucose unit of cellulose are subjected to various reactions alongside organic compounds like methyl, amine, amide, and ethyl groups, shown in Scheme 1.^28–30^ However, most of the cellulose derivatives, *e.g.*, NaCMC, methyl cellulose (MC), ethyl cellulose (EC), hydroxyethyl methyl cellulose (HEMC), and hydroxypropyl cellulose (HPC), are mainly ether-functionalized compounds, where OH functional groups present at C-2, C-3, and/or C-6 undergo dehydration to form ethers under various reaction conditions,^28,31^ shown in Scheme 2.

The obtained derivative products are more reactive and have shown improved solubility in various polar and nonpolar solvents, allowing them more applicability than the mother cellulose structure.^32^ The controllable substitution of different reactive groups within the sugar subunit allows cellulose and its derivatives to possess sufficient water solubility and viscosity in aqueous solutions. Additionally, from the overall analysis, the average number of hydroxyl groups in a single glucose moiety that is etherified makes it possible to calculate the degree of substitution.^33^

## 3. Dynamic bonding mechanisms in cellulose-based hydrogels

Cellulose, composed of linear and fibrous chains of glucose units linked by β-glycosidic bonds (C1 → C4), exhibits numerous biocompatible properties, including biocompatibility, biodegradability, and mechanical toughness.^34,35^ The presence of polar and hydrophilic functional groups, such as –OH, –COOH, and –NH_2_, facilitates the application of cellulose and its derivatives in developing hydrogels for biomedical purposes.^26^ These hydrogels are synthesized through various dynamic and reversible physical and chemical bonding mechanisms. The inherent hydrophilicity and significant intramolecular crosslinking complicate their disintegration under specific conditions.^36,37^

**Fig. 1:**
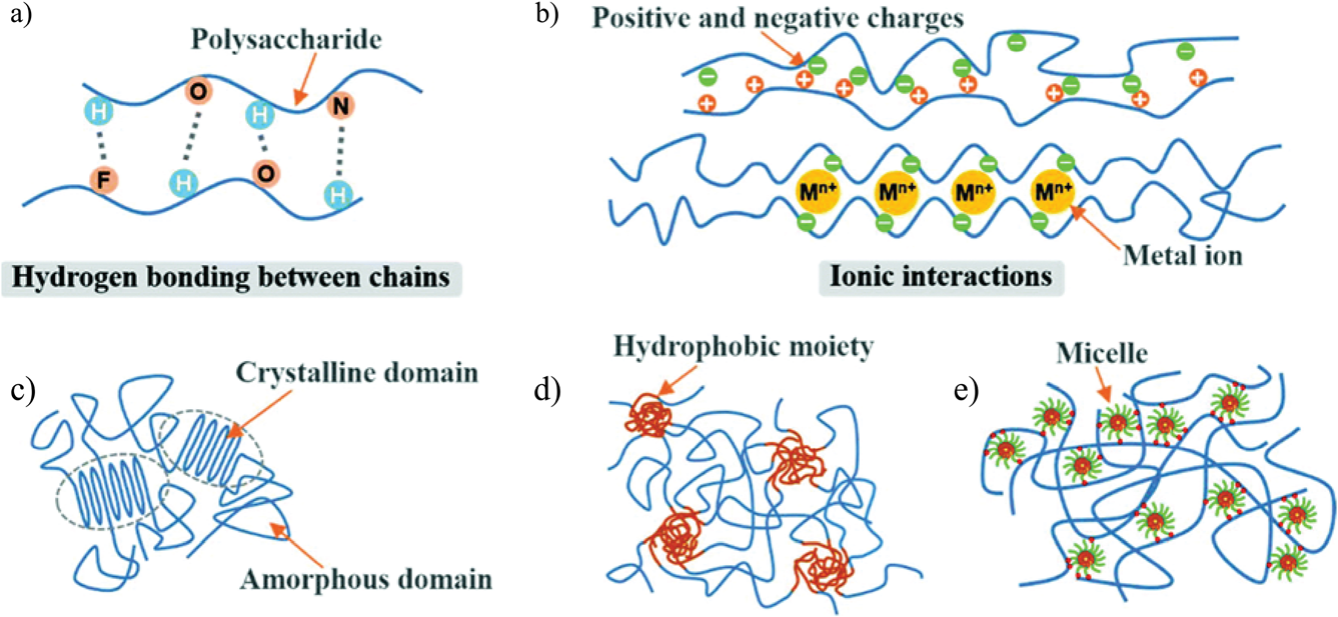
Illustrations of mechanism of various physically crosslinked cellulose-based hydrogels; (a) hydrogen bonding, (b) ionic bonding, (c) phase separation, or adsorption interactions, (d) hydrophobic interaction, and (e) micellar crosslinking. The figure is adopted with permission from ref. 38. Copyright (2020), Royal Society of Chemistry.

Recent advances in hydrogel synthesis have led to the development of physically crosslinked (PC) hydrogels that do not rely on chemical crosslinked (CC) hydrogels. Fig. 1 illustrating various physically crosslinking mechanism for the various cellulose based hydrogels.^38^ It is advisable to avoid chemical crosslinkers before clinical trials to minimize potential toxicity risks, as they may compromise the integrity of embedded compounds.^39–41^ PC hydrogels exhibit dynamic and reversible bond formation through non-covalent interactions, such as surface adsorption between adjacent polymer chains.^41,42^ Conformational optimization in PC hydrogels can be achieved through various interactions, including hydrophobic interactions, van der Waals forces, π–π stacking, hydrogen bonding, ionic interactions, and complexation.^43^ Various mechanisms of physically crosslinked hydrogels are represented in Fig. 1.

Hydrogen bonding interactions (HBI) are a widely utilized approach in the physical crosslinking of supramolecular hydrogels.^44,45^ Due to its unique orientation and adaptability, HBI significantly influences essential biological processes, including DNA recombination, molecular recognition, and protein folding.^46^ HBI occurs when a hydrogen atom interacts with a highly electronegative heteroatom, such as oxygen, nitrogen, or halogens, which possess lone-pair electrons.^47^ This interaction is particularly advantageous in supramolecular hydrogel systems, as it enhances mechanical strength, promotes flexible chain orientation, and allows for reversible crosslinking, often outperforming other non-covalent interactions.^48,49^

The mechanical strength of HBI-based gels is primarily influenced by solvent polarity, the percentage of HBI active sites, the total number of hydrogen bond donors and acceptors, and the arrangement of these functional groups.^50–52^ Consequently, the strength of hydrogen bonds can vary from highly dynamic to nearly covalent, providing extensive opportunities for the design of HBI-based hydrogels.^53^ Researchers worldwide have leveraged the potential of HBI to develop diverse polymers, exploring novel concepts related to cellulose-based hydrogels.^54^ The numerous polar functional groups in cellulose, particularly the abundant hydroxyl groups on its surface, contribute to its supramolecular structure involving HBI.^55,56^ Inspired by the complex chemistry of mussels, Wu *et al.*,^57^ introduced a method for developing durable and responsive hydrogels using microcrystalline cellulose (MCC) and konjac glucomannan. This hydrogel exhibited significant intermolecular HBI, exceptional mechanical toughness, rapid self-healing abilities, improved pH properties, and reduced initial burst release compared to KGM hydrogels, highlighting its potential as a carrier for controlled biomolecule and drug delivery.^57^

Biyani *et al.*,^58^ explored the synthesis of a supramolecular polymer composite by modifying cellulose nanocrystals (CNCs) with 2-ureido-4-pyrimidone (UPy). The resulting nanocomposite displayed robust mechanical toughness and efficient optical healing, attributed to the increased HBI crosslinking density. Innovative research has combined hydrogen bonds with various bonding techniques, such as incorporating cellulose nanofibrils (CNFs) into a polyacrylic acid (PAA) gel matrix, which significantly enhanced the hydrogel's mechanical toughness and ionic conductivity due to the physical entanglements and HBI formed between PAA and the polar functional groups of CNFs.^58^

**Fig. 2:**
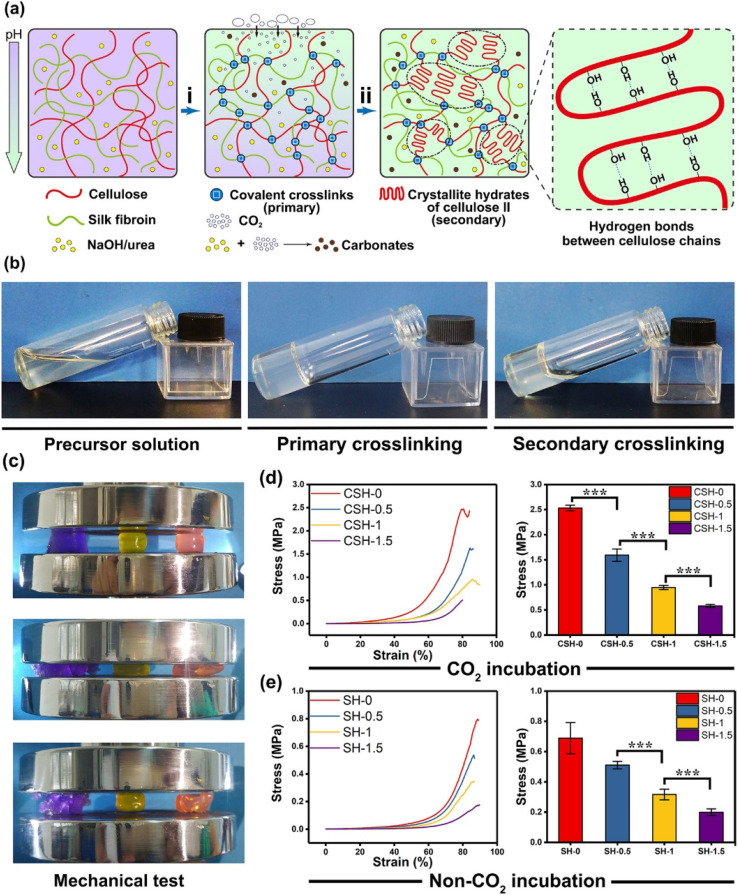
Development and analysis of highly flexible hydrogels. (a) Synthesis of cellulose/silk fibroin hydrogels (CSHs) using a two-step cross-linking approach. The initial step (i) involved the covalent bonding of cellulose and silk fibroin mediated. The subsequent cross-linking step (ii) leveraged hydrogen bonding among cellulose chains, facilitated by a CO_2_ exposure method. In this process, NaOH and urea were present in the hydrogel during the primary cross-linking phase (i), (b) images of various hydrogel samples captured at different fabrication stages, including precursor solution, after primary cross-linking, and following secondary cross-linking, (c) visual representation of mechanical performance tests on hydrogels, demonstrating superior mechanical properties in CO_2_-incubated samples (purple: hydrogels with primary cross-linking only; yellow: double cross-linked cellulose hydrogel; pink: double cross-linked cellulose and silk fibroin mediated hydrogel), (d and e) mechanical test results indicating that the strength of CSHs reached the megapascals range but decreased with increasing silk fibroin content. In contrast, SHs without CO_2_ treatment exhibited significantly lower mechanical strength compared to CO_2_-incubated CSHs. CSH-0, CSH-0.5, CSH-1, and CSH-1.5 corresponded to SF concentrations of 0, 0.5, 1, and 1.5%, respectively, while SH-0, SH-0.5, SH-1, and SH-1.5 represented equivalent SF contents in SHs. Data were presented as mean ± SD, *n* = 5, and signifies *P* < 0.001. The figure is adopted with permission from the ref. 60. Copyright © 2020 American Chemical Society.

In another approach, Gong *et al.*,^59^ developed a hydrogel with a chain-like structure composed of aligned fibrous frameworks. This design led to significant enhancements in mechanical strength through dehydration, as the alignment of polymer chains contributed to the formation of intricate fibrous structures. The natural toughness and rigidity of these cellulose hydrogels enabled them to respond to mechanical signals, aided by structural HBI, which facilitated the formation of anisotropic structures.^59^ Mao *et al.*,^60^ engineered a gel network incorporating non-covalent bonds, demonstrating chain alignment, a crystalline network structure, reversible covalent bonding, and dynamic HBI. Physical crosslinking was induced using a CO_2_ medium to synthesize double-network gels from cellulose and silk fibroin. By adjusting the pH levels, the affinities between cellulose and other polymers led to the formation of hydrogels with significantly enhanced toughness, underscoring the practical implications of HBI in developing cellulose-based responsive biomimetic polymers.^60^ To improve the mechanical robustness and hemostatic performance of hydrogel dressings, we developed a novel cellulose/silk fibroin hydrogel (CSH) by combining two natural polymers-cellulose and SF-using an innovative CO_2_-incubation method (Fig. 2). This environmentally friendly CO_2_-assisted crosslinking approach enhances the mechanical integrity of the CSH while eliminating the toxicity associated with conventional crosslinking agents. Furthermore, the resulting hydrogel exhibits superior biocompatibility, making it a promising candidate for biomedical applications.^60^

**Fig. 3:**
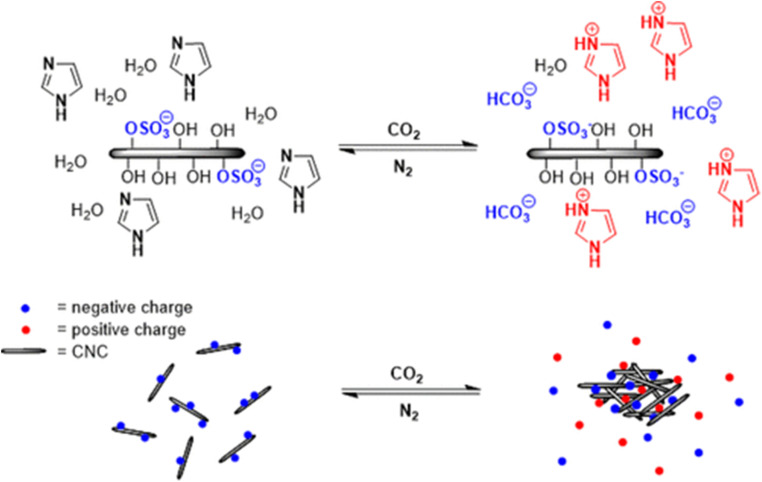
Cyclic aggregation and re-dispersion of CO_2_-responsive CNC suspensions incorporating imidazole, regulated by the addition and removal of CO_2_. The figure is adopted from ref. 64. Copyright © 2018 American Chemical Society.

Electrostatic interactions (EIs), arising from the presence of opposite charges within molecules, can be integrated into supramolecular hydrogels through the strategic design and synthesis of charged monomers or polymer chains.^61,62^ Several studies have examined cellulosic hydrogels derived from EIs and their practical applications. Langer *et al.*,^63^ designed a self-assembled hydrogel utilizing EIs between negatively charged CMC and positively charged cetyltrimethylammonium bromide, demonstrating adjustable characteristics, reduced viscosity under shear stress, and self-repairing capabilities. MacLachlan *et al.*,^64^ synthesized a CO_2_-sensitive hydrogel by incorporating a combination of imidazole and monomers into CNCs, enabling control of the gelation process without chemical initiators or additional functionalities. This discovery holds potential for carbon dioxide extraction and sensing applications. Fig. 3 illustrates the mechanisms governing the CO_2_-responsive aggregation and re-dispersion of CNCs suspensions functionalized with imidazole groups. CNCs possess a net negative surface charge due to the presence of sulfate half-ester groups, which induce electrostatic repulsion and allow them to remain stably dispersed in aqueous media. Introducing CO_2_ into the suspension prompts its interaction with water, generating carbonic acid and consequently lowering the solution's pH. This pH reduction enhances the protonation of imidazole groups (p*K*_a_H 6.95), forming hydrogen carbonate ions and imidazolium salts. The emergence of these ionic species raises the ionic strength of the medium, neutralizes the CNC surface charges, and diminishes the electrostatic repulsion between CNC particles, facilitating aggregation (Fig. 3).^64^

Huang *et al.*,^32^ engineered a biocompatible gel by integrating positively charged CNCs with negatively charged alginate (AG) in a double-network structure. The internal architecture, formed through ionic interactions between CNCs and AG, provided an efficient alternative without requiring chemical modifications.^32^ Yang *et al.*,^65^ developed a conductive hydrogel capable of self-healing and surface adhesion, where the combined effects of hydrogen bonding and EIs were critical. Tannic acid-coated CNCs served as reinforcing fillers within a doubly crosslinked network. Chang *et al.*,^66^ introduced quaternized tunicate CNCs into a crosslinked hydrogel matrix, significantly enhancing mechanical strength due to EIs between positively charged TCNCs and negatively charged PAA chains. Additionally, composite hydrogels with TCNCs exhibited pH-dependent swelling properties.^66^ Yang and Wang,^67^ developed a double-network structure utilizing quaternary ammonium group-modified β-cyclodextrin as the positively charged site and TCMC as the negatively charged site. These drug-loaded cellulosic gels, which interact through electrostatic and host–guest mechanisms, show promise for pharmaceutical applications. Liu *et al.* recently synthesized pH-responsive gels from CMC and bovine serum albumin (BSA) using an environmentally friendly method involving EIs, suggesting potential for combined chemo-radioisotope treatments for cancer.^67^

Within the category of thermo-reversible hydrogels, cellulose emerges as a prominent constituent, especially when engineered with hydrophobic functional groups.^68^ The incorporation of methyl or hydroxypropyl groups partially hinders hydrogen bond formation, enhancing the water solubility of the resulting derivatives.^69^ Gelation of cellulose derivatives occurs due to the exclusion of water from strongly methoxylated regions of the polymer. Aqueous solutions of MC exhibit unique properties, allowing reversible bond formation through hydrophobic interactions under thermal conditions.^70^ Compared to hydroxypropyl methylcellulose (HPMC), MC has a lower gelation temperature and exhibits fewer mechanical characteristics, such as mechanical stress and strain.^71^ Sekiguchi *et al.*,^72^ developed a near-infrared and small-angle X-ray scattering-induced MC-based thermos-responsive gelation system, where hydrophobic and HBI interactions contributed to gel formation. The results highlighted significant differences in gelation properties among regio-selectively methyl-substituted cellulose derivatives, indicating that gel formation results from the synergistic effects of hydrophobic interactions involving methyl groups and hydrogen bonds among hydroxyl groups, with the specific arrangement of methyl groups playing a crucial role. Subjecting the solution to repeated heating and cooling cycles ac cellulose rates the gelation of MC in cold water, particularly at higher concentrations.^72^ The gelation rate increases during the second heating–cooling cycle compared to the initial one. The practical application of MC hydrogels includes coating polystyrene dish surfaces to cultivate human embryonic stem cell (hES) clusters, facilitating the growth of embryoid bodies in suspended liquid culture. This process enhances the ionic strength and shielding effect of negatively charged CNCs, promoting gelation. The introduction of nitrogen gas into the solution reverses this process, reducing ionic strength and enabling CNC dispersion, resulting in a stable suspension.^72^ Notably, hES cells within embryoid bodies express molecular markers indicative of samples from all three embryonic germ layers, suggesting that the MC-coated medium can generate numerous hES cells clones. During HPMC synthesis, MC undergoes modification involving the addition of glycol ether groups to the glucose units of the cellulose structure, yielding a derivative with a molecular weight surpassing that of MC. Weiss *et al.*,^73^ successfully synthesized diverse biological materials using HPMC as a fundamental building block. Conversely, an electrostatic interaction-based gel system exhibits potential due to various remarkable properties, including controlled swelling, conductivity, and self-healing behaviours. The introduction of counterions as plasticizing agents into the hydrogel system fosters ionic interactions among polymers, facilitating hydrogel formation. The metal-coordinated approach involves the polymerization of a polyelectrolyte system in the presence of positively charged metal ions. Percec *et al.*,^74^ designed a neutral hydrogel with an equal number of positive and negative charges within the polymer network. Upon forming dynamic metal–ligand interactions, these hydrogels achieved a highly reversible and self-healing network that maintained stability under physiological conditions. The resultant gel exhibited a homogeneous, micro-sized porous structure characterized by well-organized chain networks. The hydrogels demonstrated dynamic properties, exhibiting dual-sensitive sol–gel transformations under varying pH and redox conditions, with hydrochloric acid (HCl) and trimethylamine (TEA) employed to modulate the solution pH. Notably, the hydrogel fractured upon the addition of 1,4-dithiol-dl-threitol (DTT) but was capable of self-healing through hydrogen peroxide (H_2_O_2_) oxidation. This phenomenon was prolonged due to the dynamic, reversible disruption and repair of acyl hydrazone and disulfide bonds within the hydrogels. Additionally, citric acid (CA) was incorporated with cysteamine dihydrochloride (CYS), which possesses disulfide bonding sites, resulting in a pH- and redox-responsive gel system.

The Diels–Alder reaction, a well-established chemical process, was utilized for dynamic bond formation, enabling these bonds to undergo reversible cleavage and reformation upon thermal stimulation.^75^ Nanocomposite gels were synthesized using maleimide-functionalized CNCs and furan-modified gelatine through a Diels–Alder process.^76,77^ Several studies reported that maleimide-terminated polyethylene glycol (PEG) and furan-modified CNCs were effective in developing strong, self-repairable polymeric gels.

**Fig. 4:**
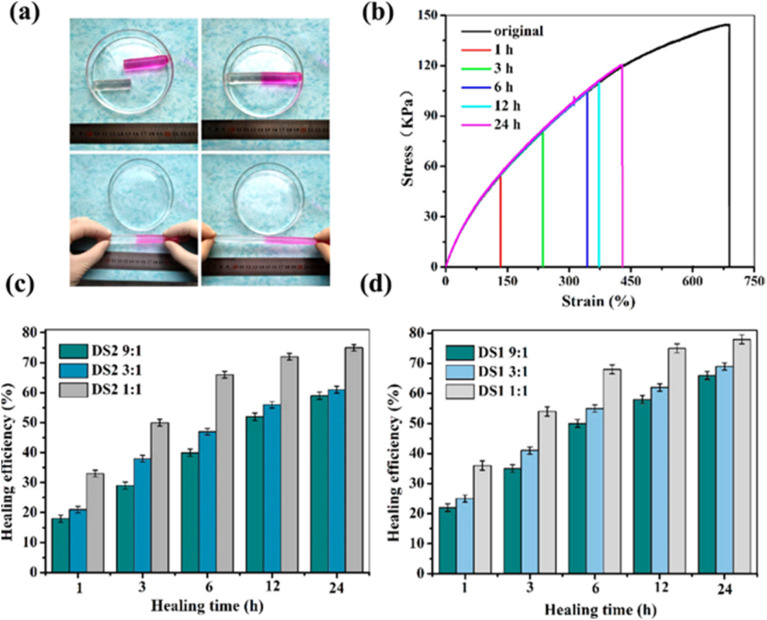
(a) Self-healing performance of hydrogel DS1 1 : 1 by direct visual inspection. (b) Stress–strain curves of the original and self-healed hydrogel DS1 1 : 1 at various healing times. (c and d) Self-healing efficiency of the hydrogel measured from tensile tests at room temperature. The figure is adopted from the ref. 78. Copyright © 2017 American Chemical Society.

Changyou Shao *et al.*,^78^ introduced a novel self-healing and self-recovering hydrogel by utilizing a furyl/maleimide pair and CNCs. In this system, CNCs function both as a reinforcing agent and a chemical crosslinker through a reversible Diels–Alder reaction. The study involved modifying CNCs with furyl groups to serve as multifunctional crosslinking agents, while PEG with maleimide terminal groups formed the polymer matrix. These components were combined to fabricate nanocomposite hydrogels capable of self-healing *via* the dynamic Diels–Alder reaction (Fig. 4).^78^ The gelation time and swelling behaviour of the hydrogels was analyzed under varying compositions. Results indicated that mechanical strength and self-healing efficiency could be fine-tuned by adjusting the substitution degree of furyl groups and the molar ratio between furyl and maleimide groups. To develop self-healing nanocomposite hydrogels, furyl-modified CNCs serve as multifunctional cross-linkers, while PEG with maleimide terminal groups functions as the polymer matrix. These components interact through a reversible DA reaction, enabling self-healing properties that can be fine-tuned by adjusting the substitution degree of furyl groups and the molar ratio of furyl to maleimide. The healing mechanism is driven by the dynamic cleavage and reformation of reversible cross-links, facilitated by thermally responsive DA bonds. This process promotes the formation of new phases at fractured surfaces, enhancing the hydrogel's self-repair ability. Moreover, a higher degree of furyl substitution combined with a lower furyl-to-maleimide molar ratio leads to an overall increase in healing efficiency over time. Since self-healing is primarily governed by DA bonds formed *via* furyl groups on CNCs and maleimide groups in PEG, a greater concentration of these bonds within the hydrogel network accelerates the repair process, ultimately improving healing efficiency.

To demonstrate self-healing, macroscopic tests were conducted, including visual observation and tensile analysis. In one test, a hydrogel sample (DS1 1 : 1) was split into two sections, with the fractured surfaces pressed together and incubated at 90 °C in an inert atmosphere. After cooling to room temperature, the merged hydrogel was subjected to tensile testing, showing no separation and confirming that the interface was robust enough to withstand applied stress. This self-healing process was attributed to the reversible cleavage and reformation of Diels–Alder bonds, which facilitated the reconstruction of the crosslinked network across the fractured regions. To evaluate the self-repairing performance, tensile properties were measured at various contact durations. Fig. 4 illustrates the stress–strain profiles of the repaired and pristine hydrogels. The data indicate that both the maximum elongation and tensile strength improved with extended healing time. For example, after 1 hour, the hydrogel achieved a fracture stress of 52 kPa, corresponding to a healing efficiency (HE) of 35%. With a 24-hour healing period, the recovered stress increased to 116 kPa, reflecting a remarkable HE of 78% compared to the original hydrogel.^78^

**Fig. 5:**
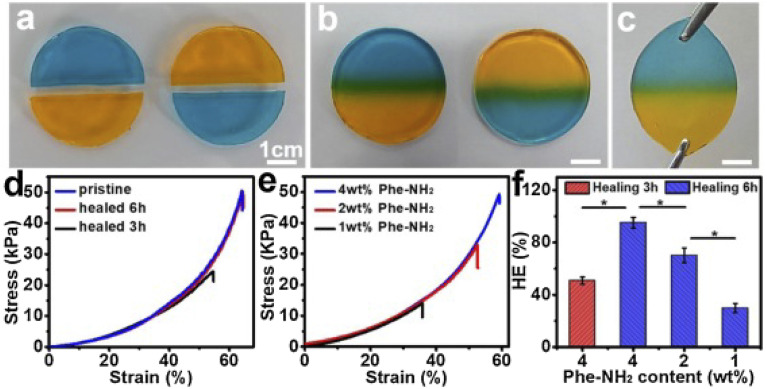
Demonstration of the self-healing behaviour of CECT-ADH/PEG-DA hydrogels at 37 °C, (a) two Gel5 disks, colored with MeO and MeB dyes respectively, were each divided into halves, (b) semi-circular halves of Gel5 with different colors were combined into a single unified Gel5 disk after 6 hours of healing, (c) the self-repaired Gel5 disk being stretched to illustrate its mechanical integrity, (d) typical compression stress–strain profiles of Gel5 before healing and after healing for 3 and 6 hours, (e) compression stress–strain profiles of hydrogels with varying Phe-NH2 concentrations following 6 hours of healing, (f) influence of healing time and Phe-NH2 content on the healing efficiency (HE) of the hydrogels after 6 hours of contact. The figure is reprinted from ref. 79. Copyright © 2020 Published by Elsevier Ltd.

Yang *et al.*,^79^ developed cellulose-based self-healing hydrogels utilizing CMC-thermoplastic hydrogel (TPH) and PEG-diacrylate (PEG-DA) *via* dynamic covalent acylhydrazone linkages under the influence of the catalysis. The self-healing abilities of the CEC-TPH/PEG-DA hydrogels are illustrated in Fig. 5.^79^ Two stained hydrogel disks were cut in half; after keeping the semicircles in intimate contact along the cut line at room temperature for 6 hours, they completely merged into an integral hydrogel disk without any external intervention. The resultant hydrogel was sufficiently strong to withstand a tensile force applied perpendicularly to the cut surface without splitting.^79^ The self-healing process was documented using optical microscopy, revealing that the two dye molecules spread across the cut surfaces and ultimately interpenetrated, resulting in a purple coloration at the boundary. The strain compression test indicated that the fracture strength of the self-healed hydrogels increased with prolonged healing time, approaching values near the original strength. This finding suggests that the self-healed hydrogels effectively restored their original mechanical properties. Additionally, healing efficiency (HE) was found to depend on both healing time and 4a-Phe content, achieving a high HE of approximately 96% at 12 hours. Consequently, upon crack formation, CEC-TPH and PEG-DA macromolecules migrated towards the damaged interface, allowing for the reformation of reversible acylhydrazone linkages and recovery of the hydrogel networks.^79^

**Fig. 6:**
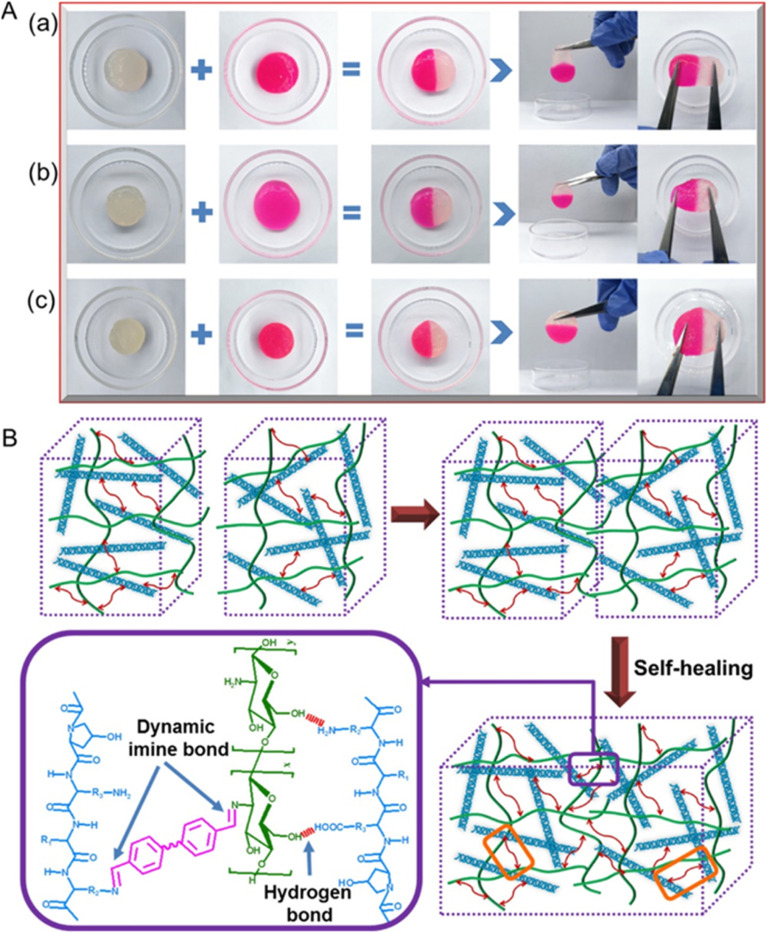
(A) Visual comparison showcasing the pristine and repaired gel samples of COL-CS hydrogels: (a) COL-CS (1 : 1), (b) COL-CS (1 : 2), and (c) CS. (B) Schematic illustrating the hypothesized self-healing mechanism. Adapted with authorization from ref. 80. Copyright © 2020, American Chemical Society.

Ding *et al.*,^80^ employed a telechelic cross-linking approach to impart pH-responsive self-healing properties to a composite hydrogel composed of chitosan and difunctional poly(ethylene glycol) (DF-PEG), as illustrated in Fig. 6. When utilized during the recovery process of rat-liver laceration, a thrombin-loaded hydrogel (CPT) applied to the liver capsule exhibited a smooth surface and vivid coloration, highlighting its potential as a highly efficient drug delivery system for treating wounds *in vivo*.

**Fig. 7:**
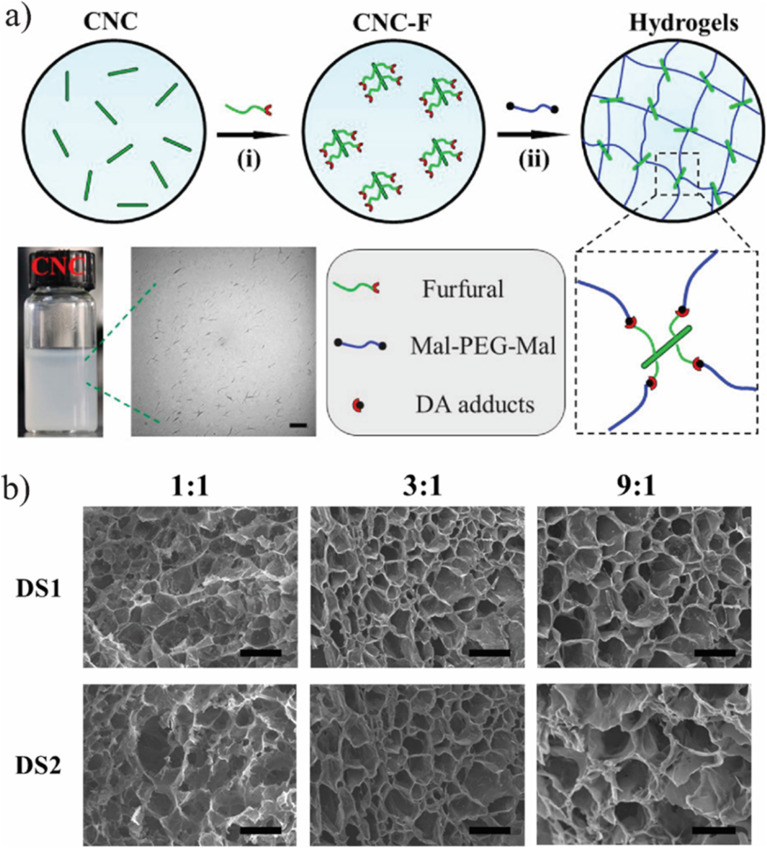
(a) Synthetic pathway for fabricating self-repairing CNC-PEG nanocomposite hydrogels, and (b) SEM images of cross-sectional views of CNC-PEG hydrogels. The figure is reprinted from ref. 78. Copyright © 2017 American Chemical Society.

In another study, Shao *et al.*,^78^ developed self-healing cellulose-based hydrogels characterized by high toughness and resilience, utilizing furyl-modified CNCs and maleimide end-functionalized PEG through a thermally reversible covalent Diels–Alder click reaction (Fig. 7).^78^ The hydrogels demonstrated remarkable mechanical properties, exhibiting a fracture elongation of up to 690% and a fracture strength of 0.3 MPa at a strain of 90%. The self-healing capability of these composite hydrogels was assessed *via* tension tests, revealing an efficiency of 78%. The researchers also constructed a tough and self-healing gel by establishing synergistic multiple coordination bonds among tannic acid (TA)-coated CNCs, PAA chains, and Al^3+^ ions within a covalent polymer network. The incision of the PAA-TA@CNC-Al^3+^ gel exhibited automatic self-healing, disappearing almost entirely within 30 minutes. The self-healed gel maintained sufficient strength to support itself and stretch without failure at the interface (Fig. 7A). The fracture stress of the healed gel significantly increased with extended healing time (Fig. 7B), reaching an equilibrium state with an HE of up to 92% after 30 minutes of healing. The HE was related to the TA@CNC content, with an increase in TA@CNC concentration enhancing the HE of the gel after 30 minutes (Fig. 7C), underscoring the importance of dynamic TA@CNC motifs for reversible rearrangement during the self-healing process. Microscopic self-healing behaviour of the gel was further validated through rheological experiments, which examined the breakup and reformation of the gel network (Fig. 7D). As anticipated, switching the strain from 100% to 1% at a fixed frequency allowed for immediate recovery of the gel-like character (*G*′ > *G*′′) without significant decrease across multiple recovery cycles. This sol–gel transition, accompanied by complete recovery of the gel network following disruption, was attributed to the reconstruction of reversible ionic coordination complexation within the gel network system.

Chemical crosslinking is commonly employed to enhance the structural stability and desirable swelling properties of hydrogels derived from cellulose.^81^ Bi-functional molecules act as crosslinking agents, facilitating the formation of covalent bonds between cellulose or its derivatives and other polymer chains, thereby establishing a hydrophilic network.^22^ Sannino *et al.*,^82^ developed superabsorbent hydrogels based on cellulose by crosslinking CMC and hydroxyethyl cellulose (HEC) with divinyl sulfone (DVS). These hydrogels exhibited exceptional water absorption capacity, particularly under varying pH and ionic strength conditions. Moreover, these cellulose superabsorbent hydrogels were designed to address swelling by effectively extracting excess water from tissues. HPC hydrogels, synthesized under specific thermal conditions within an integrated phase system, demonstrated a nonporous structure throughout the biphasic system; however, alterations in conditions triggered the formation of a microporous structure within the hydrogels.

Hirsch and Spontak^83^ investigated the swelling capacities and dynamic mechanical properties of these pre-formed gels, analysing how they varied with changes in crosslinking temperature. The correlation between temperature and HPC gel swelling properties was limited. Conversely, the modulus of microporous HPC gels could be enhanced by extending the crosslinking duration. Thermally responsive hydrogels were synthesized through the crosslinking of HPC with PEG ether. Marsano *et al.*,^84^ developed hydrogels exhibiting swelling behaviour at lower temperatures (20 °C) and contraction activity at higher temperatures (60 °C). HPC hydrogels, fabricated through crosslinking with epichlorohydrin (ECH) and ammonia, demonstrated a remarkable capacity for absorbing anionic dyes, with a maximum adsorption capacity of 2478 g kg^−1^ at ambient temperature and a pH of 3.96.^84^

One crucial property of hydrogels is the SR, which can be manipulated through various means, including modifications in crosslinking length, alterations in the molecular weight of the gel systems, or the incorporation of filler materials to occupy the spaces between macromolecular chains,^85,86^ when DVS is used as a crosslinker. For gel structures to be suitable for applications in medicine, food, and biomaterials, the crosslinkers employed in their synthesis must be non-toxic. This is an essential area of research that scientists actively pursue, focusing on developing new biocompatible crosslinkers to create hydrogels based on cellulose.

Since carbodiimide molecules do not promote crosslinking, water-soluble carbodiimide is often utilized to enhance the biocompatibility of cellulose hydrogels through the crosslinking process.^87^ Furthermore, it is feasible to convert them into urea derivatives, which are less harmful to cellulose and can be easily washed out of the polymeric network. The SR of hydrogels is influenced by the chemical composition of CMC and HEC as well as the dehydration process.^27^ Dehydrated hydrogel pellets are designed for oral administration. According to Sannino *et al.*,^88^ in the acidic pH of the stomach, these pellets will swell, inducing a sensation of fullness before being excreted in the faces. Demitri *et al.*,^89^ successfully demonstrated the development of hydrogels composed of HEC and CMC, synthesized using CA as a crosslinking agent. This method offers several advantages, including reduced toxicity and cost-effectiveness compared to previous crosslinking agents. The proposed esterification process involves the synthesis of an anhydride intermediate, which is hypothesized to play a critical role in the interaction between cellulose and CA. The swelling degree is influenced by the duration of the active reaction and the concentration of CA; at a CA concentration of 3.75% (w/v), the hydrogel exhibits an SR of 90.

Various bonding mechanisms can influence the physical and chemical properties of cellulose-based hydrogels.^70^ The choice of suitable hydrophilic or hydrophobic groups, as well as the selection of compatible polymer matrices, plays a pivotal role in achieving desirable properties for specific biomedical applications. Continued exploration of cellulose-based hydrogels is essential to unlock their full potential, providing novel insights for advancing biocompatible materials and enhancing their performance across diverse biomedical fields.

## 4. Swelling properties of cellulose-based hydrogels

**Fig. 8:**
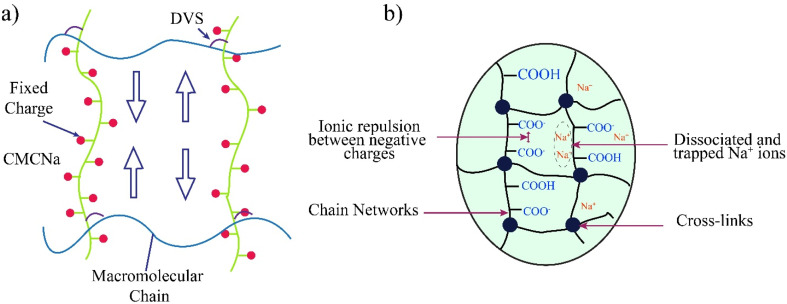
Swelling mechanism of hydrogels under (a) changing the cross-linking length to adjust the gel mass size. The idea of this figure is taken from the ref. 93. Copyright © 2006 Royal Society of Chemistry. And (b) ionic conditions. The idea of this figure is taken from the ref. 94. Copyright © 2014 Royal Society of Chemistry.

The swelling mechanism of polymeric hydrogels reveals the underlying processes responsible for water absorption and retention within the three-dimensional network when these materials are exposed to a solvent.^90^ The chain network of hydrogels contains charged polar functional groups, which primarily contribute to the hygroscopic properties of this advanced polymer. Several studies reveal that charged particles within the gel matrix generate repulsive forces and osmotic pressure (OP), which drive the swelling phenomenon.^91^ Specifically, negative charges within the network exert repulsive forces that facilitate the expansion of the gel structure.^92^ However, the presence of crosslinked polymers in hydrogels mitigates disintegration, thereby limiting excessive swelling, as illustrated in Fig. 8(a).^93^

Hydrogels can absorb and retain significant quantities of water, contingent upon the nature and extent of cross-linking as well as the charge densities of their networks.^95,96^ The swelling degree (SD) quantifies the amount of water absorbed by a hydrogel. This property is critical for the *in vivo* application of hydrogels, as excessively swollen materials may disintegrate prematurely during wound healing or cause tissue damage in confined anatomical spaces.

The degree of swelling notably influences the diffusion of bioactive molecules or drugs from the swollen gel matrix. Given that hydrogels can absorb and retain water, they exhibit high swelling degrees and molecular diffusion rates.^97^ To assess the SR, hydrogels undergo various drying processes. Subsequently, the specimens are immersed in an adequate volume of water at room temperature until they reach an equilibrium state. During this period, the hydrogels undergo filtration, and their weights are measured. Equilibrium is achieved when the weight of the swollen hydrogels stabilizes. Thus, the equilibrium swelling degree (ESD) can be calculated using eqn (1).^98,99^

1 ESD = (*W*_s_ − *W*_d_)/*W*_d_

At equilibrium, the weights of dry and swollen gels are represented as *W*_d_ and *W*_s_, respectively. Various factors, such as crosslinking density, type of crosslinking, solvent properties, and the interaction between the polymer and solvent, can influence swelling behaviours and the extent of swelling.^100,101^ Water acts as a softener or plasticizer in a hydrophilic polymer network structure. The swelling degree of gels can be explained using the Flory–Rehner equilibrium hypothesis (eqn (2)),^101,102^ which is mainly based on the Gibbs free energy concept, to analyze the swelling behaviour of the obtained hydrogels.^103,104^

2 Δ*G*_total_ = Δ*G*_mix_ + Δ*G*_el_

The variables denoted as Δ*G*_total_, Δ*G*_mix_, and Δ*G*_el_ represent the free energy contributions from the mixing enthalpy and elastic retractile stresses within the polymer network. Initially, the change in Gibbs free energy for mixing, denoted as Δ*G*_mix_, has a substantially negative value. In contrast, the change in Gibbs free energy for an electrochemical process denoted as Δ*G*_el_ is positive but with a magnitude smaller than that of Δ*G*_mix_. Therefore, the combined impact of these factors has a negative outcome (Δ*G*_mix_ + Δ*G*_el_ < 0). During this stage, swelling starts, leading to the migration of solvent molecules into the polymer network. Both are raised until they reach zero magnitudes during swelling, as shown by the formula |Δ*G*_mix_| = |Δ*G*_el_|. This results in zero total free energy, or Δ*G*_total_ = Δ*G*_mix_. The development of hydrogel adhesives for wound closure involves essential elements, including the density of cross-linking (*ρ*_x_), the polymer volume fraction when swollen (*ν*_2,s_), the molecular weight of polymer chains between neighbouring cross-links (*M*_c_), and the mesh size (*ξ*). These characteristics play a vital role in determining the mechanical and diffusive properties of the material upon application to a wound site. As a result, there is no longer any driving force for the swelling, which causes it to cease and reach equilibrium.

However, the quantitative analysis of swelling behaviour can be obtained by using following equations,^96^3
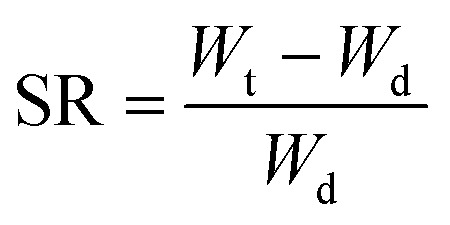
where *W*_t_ and *W*_d_ are the weights of the swollen and dry hydrogel, respectively.^96^

By using this mathematical relationship, for example, Rahman *et al.*^95^ synthesized dicarboxylate nanocrystalline cellulose (DCNC) from microcrystalline cellulose (MCC) through acid hydrolysis and selective periodate oxidation. The polyelectrolyte (PE) nature of DCNC was analysed by measuring water uptake using a customized ion exchange system. Two DCNC samples with carboxylate contents of 9.65% and 33.33% exhibited increasing water absorption over time, reaching equilibrium. The higher carboxylate content (33.33%) resulted in the highest water uptake (≈60 times its weight), while the lower content (9.65%) absorbed ≈30 times its weight. In contrast, TEMPO-oxidized NCC (9.72% carboxylate content) absorbed only five times its weight, indicating that oxidation position significantly influences water uptake. DCNC exhibited nearly ten times greater equilibrium water absorption than TEMPO-oxidized NCC, confirming that C-2 and C-3 functionalization enhances water uptake performance.^95^ Another important example, Nguyen *et al.*,^105^ reported “dip-catalyst” hydrogels for methylene blue (MB) dye removal, where the catalyst is immobilized and uniformly distributed within the hydrogel matrix. TiO_2_ served both as a crosslinker and photocatalyst, enabling structural integrity and catalytic activity. The study examined crosslinking density by analyzing the swelling behaviour of cellulose-based hydrogels with different TiO_2_ crosslinkers. When immersed in distilled water at room temperature, all hydrogel samples exhibited a rapid increase in swelling capacity within the first 60 minutes, driven by OP and electrostatic repulsion between negatively charged groups. Beyond this period, the swelling rate gradually declined, reaching equilibrium after approximately 150 minutes. The cross-linked polymer network restricted excessive solvent absorption, thereby limiting the hydrogel's maximum swelling capacity.^105^

### 4.1. Structural properties

Several critical factors influence the SR properties of cellulose-based hydrogels, particularly in their application as structural biomaterials, such as wound dressings.^106^ The primary objective is to evaluate their effectiveness for these applications. The parameters include the optimal density of the polymer chain networks, denoted as *ρ*_x_, which represents the proportion of polymer volume when fully swollen. These factors significantly affect the mechanical and diffusion properties of the hydrogel once it interfaces with the wound. Understanding and controlling the density of chain networks is essential, as it directly influences the gel's mechanical strength and its ability to adhere to the wound surface.^107^ Furthermore, the fully swollen polymer volume proportion affects the gel's capacity to absorb and release therapeutic agents, thereby promoting wound healing.^108^ Consequently, optimizing these parameters is vital for ensuring the successful application of cellulose hydrogels as effective wound dressings in biomedical contexts.

The equilibrium-swelling (ES) theory and rubber-elasticity (RE) theory are key approaches for understanding the bonding and crosslinking characteristics of polymer network systems.^109,110^ The ES theory primarily focuses on the gel network's ability to integrate and describes how these networks interact within an aqueous environment. This analysis provides valuable insights into the structural parameters, particularly regarding the mechanical and crosslinking behaviour of the chain network. Conversely, the RE theory examines the elastic properties of the gel matrix and its ability to maintain structural integrity under applied stress.^111,112^ Upon the removal of stress, the theory elucidates how the matrix reverts to its original state, which is fundamentally dependent on the nature of crosslinking within the gel matrix.

Analysing material properties through RE is significant for investigating correlations and structural parameters. Numerous equations have been developed in the scientific literature to compute these parameters, which vary based on the gel structure and the methodologies employed for their determination. For example, in a study examining the deformation of a crosslinked polymer gel under stress, researchers applied varying stress levels and utilized rheological techniques to assess changes in the network structure.^107,113^ By observing how the gel's structure altered under stress and subsequently returned to its initial form, they gleaned insights into the type and strength of crosslinking within the gel matrix. This information was instrumental in understanding the material's response to external forces and enabled the determination of critical structural parameters, such as elasticity and stability.

Moreover, this knowledge served as a foundation for further optimization and development of the gel material. The researchers successfully modified the gel formulation and crosslinking process to enhance both elasticity and stability, resulting in a more robust gel capable of withstanding higher stress levels without significant structural changes. These advancements opened avenues for diverse applications, including biomedical engineering, where the gel could serve as a scaffold for tissue regeneration or as a drug delivery system. The researchers remained optimistic that ongoing optimization efforts would yield even more versatile and efficient gel materials in the future.

### 4.2. Water vapor transmission rate

Hydrogels possess a unique ability to absorb and retain water molecules within their matrix while also desorbing them under varying atmospheric conditions.^114,115^ Due to their properties closely resembling those of soft natural tissues, hydrogels are widely utilized in the biomedical field, particularly in wound treatment.^1,116–118^ In an ideal hydrogel application for wound care, the hydrogel encapsulates the wound surface, effectively absorbing wound exudates and preventing dehydration. This mechanism significantly improves the wound healing process compared to conventional treatment methods.

When applied to a wound, cellulose-based hydrogels absorb exudates such as pus and fluid, thereby maintaining a moist environment conducive to faster healing.^119^ Additionally, cellulose-based hydrogels can be easily removed from the wound, minimizing pain and discomfort during dressing changes and enhancing patient comfort. Furthermore, these hydrogels exhibit antimicrobial properties, reducing the risk of infection and associated complications. Overall, cellulose-based hydrogels represent a promising approach in wound treatment, not only accelerating the healing process but also improving patient comfort and minimizing infection risks.^120^

The rate of water vapor transmission (*R*_WVT_) is a widely employed technique for quantifying the amount of water vapor released from the hydrogel network. This measurement is obtained by analysing the vapor process across a unit surface area of hydrogels (cm^2^) under controlled conditions, including a fixed time, specific temperature, and constant humidity level. The *R*_WVT_ is expressed in grams per square meter per hour (g m^−2^ h^−1^) using the following formula:^121^

4 *R*_WVT_ = *t*·*A*/*G*

The weight change (*G*) is measured in grams, the time (*t*) is expressed in hours, and the tested surface area (*A*) is denoted in m^2^. For instance, wound treatments exhibiting an *R*_WVT_ ranging from 80 to 100 g m^−2^ h^−1^ are effective in maintaining moisture within the wound, thereby facilitating swift recovery.

In a standard procedure, a hydrogel is applied to the opening of a sealed cylindrical plastic container filled with purified water to prevent vapor escape. This setup is maintained at 37 °C with controlled humidity levels and monitored periodically by weighing. A graph representing weight loss over time is generated, and the *R*_WVT_ is calculated by dividing the slope of this curve by the tested surface area. In a wound healing study, a hydrogel with a known *R*_WVT_ is applied to the surface of a wound of a defined area.^122–124^ The wound is then covered with a transparent film to prevent moisture loss and placed in a controlled environment. By periodically weighing the wound and monitoring weight loss over time, the *R*_WVT_ can be determined using the slope of the weight loss curve divided by the wound's surface area (*A*). The *R*_WVT_ is a critical parameter for assessing the rate at which the hydrogel releases moisture or other substances into the wound.^124^ This information is essential for understanding the healing process and evaluating the hydrogel's efficacy in promoting wound healing. Additionally, the *R*_WVT_ can be utilized to compare various hydrogel formulations, aiding in the selection of the most appropriate option for wound care. Ultimately, determining the *R*_WVT_ offers valuable insights into the performance and therapeutic potential of hydrogels in wound healing applications.

### 4.3. Total charge balance stoichiometry

Chunyu Chang *et al.*,^125^ broadly discuss the total charge balance stoichiometry (CBS) of as-prepared hydrogels underwent examination through theoretical and experimental approaches. When reaching the equilibrium of the charge balance, it is expected to be fulfilled.^125^

5 *Q*_+_ = *Q*_−_

*Q*_+_ and *Q*_−_ represent the tally of positively charged and negatively charged networks in the gel. In each sugar unit of quaternized cellulose (QC) and CMC (given that DS < 1), the hydroxyl group can be replaced by either (CH_3_)_3_N^+^ or –COO^−^. As a result, the average mass associated with each sugar unit in QC (*M*_q_) and CMC (*M*_c_) may be estimated using the following equations:

6 *M̄*_q_ = *S*_q_*M*_1_ + (1 − *S*_q_)*M*_0_

7 *M̄*_c_ = *S*_c_*M*_2_ + (1 − *S*_qc_)*M*_0_

*S*_q_ and *S*_c_ represent the distinct structural properties of both monomers. *M*_1_, *M*_0_, and *M*_2_ denote the individual molecular weights of sugar units that have been altered with quaternary ammonium, modified with a carboxymethyl group, and remain unmodified, respectively. Consequently, the equation illustrates the *ζ*-potential of the QC/CMC solution concerning the QC/CMC mass ratio. The hydrogel network's count of positive or negative charges is determined by this parameter.8
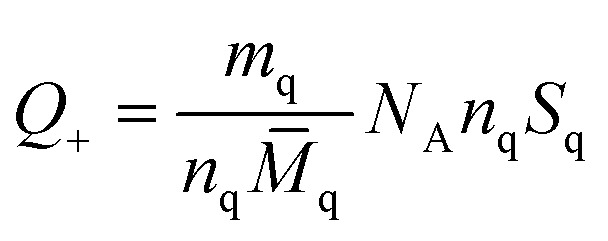
9
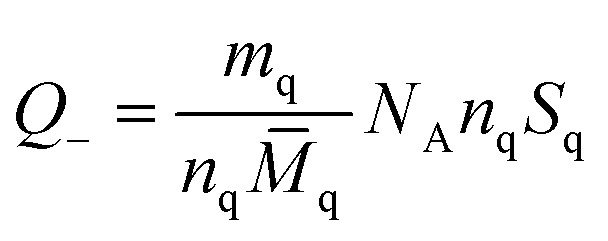


The masses of QC (*m*_q_) and CMC (*m*_c_) used in hydrogel preparation, along with their respective glucose units (*n*_q_ and *n*_c_), were considered alongside Avogadro's number (*N*_A_). The determined weight ratio of QC to CMC (*m*_q_/*m*_c_) was 1.5 upon achieving charge equilibrium (*Q*_+_/*Q*_−_).^125^

### 4.4. Effect of crosslinking materials for cellulose-based hydrogels

The synthesis of hydrogels is heavily influenced by the selection and role of crosslinking materials (CLMs).^126,127^ The primary challenge in this process lies in constructing a three-dimensional network through various synthetic methodologies, including physical crosslinking (PC), chemical crosslinking (CC), and other interactions.^128^ Chemical bonding facilitates the aggregation of adjacent chains into a continuous, interconnected network.^129,130^ CLMs can be derived from both natural and synthetic sources. Depending on the cellulose derivative employed, a variety of CLMs, such as ECH, aldehyde-functionalized materials, carboxylic acids (COOH), and urea derivatives, may be utilized in hydrogel fabrication.^131,132^ It is crucial to acknowledge that certain reagents, particularly aldehydes, pose risks in their unreacted forms. Despite thorough washing with distilled water to remove unreacted chemicals following crosslinking, it is vital to select non-toxic CLMs to ensure the biocompatibility and environmentally friendly production of the final hydrogel.^133^

Numerous studies have explored the advancement of polymeric gels utilizing CMCNa and incorporating natural CLMs.^134,135^ The production of these hydrogels often involves genipin (GP) and CA as natural crosslinking agents.^136^ GP, derived from the gardenia fruit, is a favourable alternative to dialdehyde crosslinkers due to its excellent biocompatibility.^136^ This inherent catalyst fosters the formation of covalent bonds between the gel and biological or natural tissues, such as chitosan (CS) and gelatin. Notably, GP exhibits a toxicity threshold that is 10 000 times lower than that of the commonly used glutaraldehyde (GA), which minimizes hazardous effects during hydrogel crosslinking. Muhammad *et al.,*^137^ reported the successful synthesis of a gel by combining κ-carrageenan (κC) with CMCNa using GP as a CLM. The highest SR was observed in hydrogel beads composed of a κC ratio of 90 : 10, with decreasing swelling efficiencies noted for 80 : 20, 70 : 30, and 60 : 40 ratios. This phenomenon can be attributed to the increased weight of carrageenan, which enhances the number of counterions (SO_3_^−^) in the solution, thereby strengthening the electrostatic attraction between these groups. The elevated OP resulting from the higher concentration of SO_3_^−^ ions facilitate polymer expansion.^137^

The swelling behaviour of the beads was assessed in both an acidic solution at pH 1.2 and a neutral medium at pH 7.4.^138^ Interestingly, most bead compositions exhibited greater swelling at pH 7.4 compared to pH 1.2, with SRs of 109% and 100% for the 70 : 30 bead ratio, respectively.^139^ The diminished ionization of carboxymethyl groups leads to the conversion of COONa to COOH at lower pH levels. Consequently, the ionization of carboxylic groups intensifies with increasing pH, promoting bead swelling and network repulsion. The 70 : 30 bead ratio was selected for further investigation, as the 80 : 20 and 90 : 10 ratios, despite exhibiting greater swelling, proved unsuitable for bead development due to practical synthesis challenges.^139^ Recent studies have demonstrated the effectiveness of CA as a highly compatible crosslinker in hydrogel research. Demitri *et al.*^89^ successfully synthesized hydrogels using CA, with SR analysis showing that CMCNa crosslinked with 10% CA exhibited superior swelling behaviour compared to HEC at the same CA concentration. However, at 20% CA, the swelling behaviour of HEC-based hydrogels became comparable to that of CMCNa-based hydrogels. This suggests that CA reacts more rapidly with HEC than with CMCNa, likely due to reduced steric hindrance, allowing for faster crosslinking. Gorgieva and Kokol discovered that the swelling capacity of CA-crosslinked CMCNa/HEC gels increased with higher concentrations of CMCNa.^140^ Hydrogels with a CMCNa/HEC ratio of 3 : 1 exhibited a notable swelling increase of approximately 10–20% compared to those with a 1 : 1 ratio. Conversely, hydrogels with elevated HEC concentrations showed decreased stability due to a reduction in crosslinking points, influenced by a high degree of substitution leading to fewer OH groups compared to CMCNa. At pH levels ranging from 6.25 to 6.5, the ionization of carboxylic acid groups (COO^−^) was noted, corresponding to the cellulose p*K*_a_ value of 4.6. Changes in pH instigated a breakdown of hydrogen bonding interactions (HBIs), attributed to the repulsion among macromolecules and water absorption. It is noteworthy that the hydrogel with a molar ratio of 1 : 1 exhibited fewer HBIs than the hydrogel with a 3 : 1 ratio. Additionally, the CMCNa/HEC hydrogel displayed a reduction in HB content with a higher CA crosslinker concentration (5.75% w/w) compared to 3.75% (w/w) CA. The response to pH changes was immediate; a 3.75% (w/w) CA concentration elicited greater swelling in alkaline conditions than in acidic environments. Gels with a higher pH and abundant COO^−^ groups experienced increased ionic repulsion, resulting in the expansion of the gel network and enhanced water absorption.

Durpekova *et al.*,^141^ investigated a hydrogel composed of CMCNa and HEC, with CA acting as the crosslinker. The resultant gel exhibited superior swelling behaviour when exposed to water compared to individual CMCNa or HEC hydrogels at equivalent CA concentrations. However, the incorporation of CA diminished the stability of the HEC-based hydrogel, reducing its absorption capacity. The limited crosslinking efficiency of HEC can be attributed to its higher degree of substitution compared to CMCNa. As a polyelectrolyte, CMCNa exhibits pH sensitivity and ionic strength, enhancing swelling through the Gibbs–Donnan effect and increasing OP. This elevated OP mitigates the ionic strength of the external solution, facilitating water ingress into the hydrogel.^141^ Nonetheless, CMCNa's poor crosslinking efficiency results from ionic repulsion among charged polyelectrolyte chains, favouring the formation of intermolecular over intramolecular crosslinks.

Numerous investigations have indicated that the monomer ratio and the manipulation of CLM concentration significantly affect the degree of swelling.^142^ An elevated concentration of CA in the solution mixture, resulting from enhanced crosslinking density, corresponded to a decrease in water uptake. Conversely, hydrogels with inadequate crosslinking percentages exhibited poor formation due to insufficient crosslinking.^143,144^ CMCNa/HEC (3 : 1) demonstrated an increased absorption capacity with a CA weight percentage of 5.75% in water-prepared samples, with comparable results observed in whey-prepared samples at a CA concentration of 5%.^89^ The gel synthesized at pH 4.5 displayed the most favourable SR. The introduction of whey acid (WC), a low-protein acid, as an alternative to distilled water in gel synthesis, effectively repurposed waste from the dairy industry. The water uptake capability of WC/cellulose-based hydrogels was remarkably high (1000–1700%), comparable to hydrogels synthesized with distilled water. Various pH environments were examined to assess their influence on the swelling behaviour of WC/cellulose gels. The gel reached maximum swelling at pH 7.2 in distilled water and at pH 10.0 (994%) in saline solution. A significant reduction in swelling was observed when pH decreased to 2.5 due to the protonation of COOH groups. Above the p*K*_a_ value of 4–5 for COOH groups, deprotonation occurred, leading to ionic repulsion among the negatively charged groups within the hydrogels and enhancing swelling performance. Heat application to the mechanism of CA action facilitates the removal of water from the carboxylic acid group, resulting in the formation of a cyclic anhydride.^145^ This cyclic anhydride of CA then esterifies by linking with hydroxyl groups on cellulose. According to Demitri *et al.*,^89^ dehydration of carboxylic acid to cyclic anhydride initiates at 60 °C, while degradation occurs at 160 °C. CMCNa demonstrates thermal stability below 100 °C but degrades at elevated temperatures. Therefore, the optimal temperature for crosslinking CMCNa with CA is identified as 80 °C.

Zhang and Qiao^146^ developed a polymeric gel using CMCNa as a monomer and ECH as a crosslinker. The addition of superabsorbent polymers (SAPs) to soil has been shown to reduce water evaporation and percolation. However, the as-prepared SAPs failed to meet necessary requirements due to challenges with consistent water uptake and salt tolerance. This study explored the effects of valence cations, specifically sodium, calcium, and Al^3+^ ions, on the structural modifications of ECH-crosslinked hydrogels based on CMCNa. Results indicated that the presence of additional COOH groups enhanced water absorption capacity, with a hydrogel containing 5% CMCNa and 3% NaOH demonstrating an exceptional water absorption capacity of 969.0 g g^−1^ in deionized water.^146^ However, increased cation valence in solution reduced hydrophilicity and salt resistance. The interaction between the COOH group and the Na cation resulted in proton replacement, while Ca-ion interactions involved bidentate bridging chelation, with Al^3+^ ions exhibiting tridentate chelation.

**Fig. 9:**
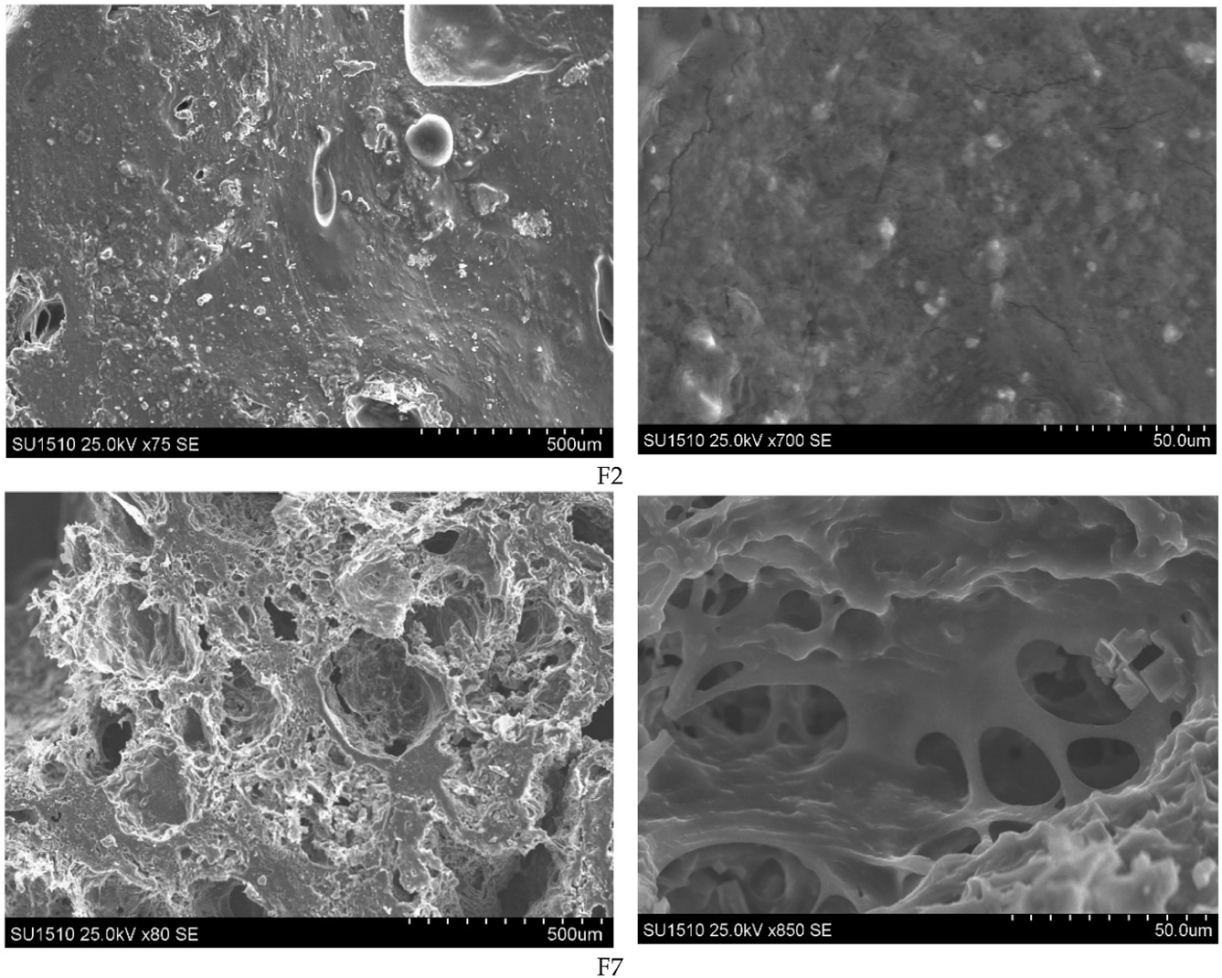
SEM images of the AG/CMC films with different resolutions. The figure is adopted from the ref. 148. Copyright 2021, MDPI.

Although the inclusion of polyvalent cations stabilized the COOH group, it concurrently hindered water uptake due to reduced swelling capacity.^147^ Following this study, Peptu *et al.*,^148^ developed a hydrogel using ECH crosslinking, where the stabilization of the COOH group by polyvalent cations also led to diminished swelling capacity. They produced an ECH-crosslinked hydrogel exhibiting excellent superabsorbent characteristics with a supreme SR of 1273% at a 1 : 1 AG molar ratio and a 6.6% monomer mixture with 0.75 mL ECH. This high SR was attributed to the porous structure observed in scanning electron microscopy (SEM) images (Fig. 9).^148^ In contrast, a sample with the same molar ratio but a higher ECH concentration (3 mL) exhibited a lower SR of 362%, likely due to an enlarged network structure resulting from excessive ECH crosslinking. This highlights the importance of the concentrations of monomers and CLMs in influencing the SR.

The swelling behaviour in a polyelectrolyte system is driven by the combined effects of Donnan OP and the elasticity of the polymeric chain network. The OP increases steadily with the number of active molecules and charges but decreases at higher ionic strengths. This relationship between OP and ionic strength is contingent upon the number of ions present in the system. At lower ionic strengths, repulsion among charged molecules prevails, with Donnan OP dominating the swelling process.^149,150^ Conversely, an increased ionic presence at higher ionic strengths screens charged molecules, reducing repulsion and consequently lowering Donnan OP. Understanding and managing the swelling behaviour of polyelectrolyte systems hinges on this interaction between Donnan OP and ionic strength.^149,150^Table 1 represents various crosslinking mechanism for various cellulose based hydrogels.

**Table 1:** Different types of crosslinking mechanism of cellulose-based hydrogels^a^

Hydrogels	Synthetic method	Crosslinker	Crosslinking mechanism	Ref.
Cellulose–bentonite hydrogel	Crosslinking	ECH	Electrostatic interaction	151
CMC-based hydrogel	Inverse suspension crosslinking	ECH	Electrostatic interaction	152
Chitosan and cellulose-based hydrogels	Freeze-dried		Electrostatic and hydrogen bonding	153
CMC-based hydrogels	γ-irradiation	Cu^2+^	Chelation	154
CMC–AM–GO-based hydrogels	Radical polymerization	GO	Covalent and electrostatic	155
BC		SA	Hydrophobic interaction	156
Cellulose	Freeze thawing	HAP	Hydrogen-bonding	157

a ECH: epichlorohydrin, CMC: carboxymethyl cellulose, AM: acrylamide, GO: graphene oxide, BC: bacterial cellulose, SA: sodium alginate, HAP: hydroxyapatite.

### 4.5. Effect of ionic strength on swelling behaviour

**Fig. 10:**
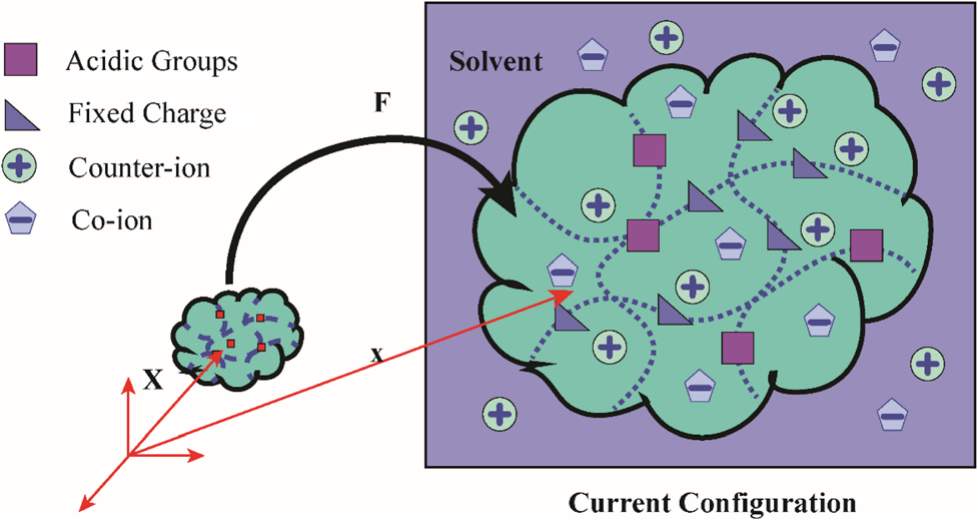
Schematic of the reference and current configurations of a pH-sensitive hydrogel with an arbitrary shape undergoing deformation due to external stimuli. The idea of the diagram is taken from ref. 158. Copyright© 2020, Elsevier B.V.

Three main components of a salt-responsive hydrogel are typically its three-dimensional structure, interstitial fluid, and many ionic groups (Fig. 10).^158,159^ However, the pH-sensitive gel networks, including the primary and acidic groups BOH and AH. A^−^/B^+^ and the related conjugate base/acid H^+^/^−^OH are formed when some acidic or basic groups dissociate when the solvent penetrates the gel.^159^

The fixed charge, denoted as the conjugate base/acid, arises from this process, contributing to a network-bound charge. Importantly, these reactions are reversible.

10 AH ↔ A^−^ + H^+^

11 BOH ↔ B^+^ + OH^−^

Chemical equilibrium is achieved when the concentrations of the reactive chemicals AH/BOH, A^−^/B^+^, and H^+^/OH^−^ remain constant over time. The dissociation constant is,12
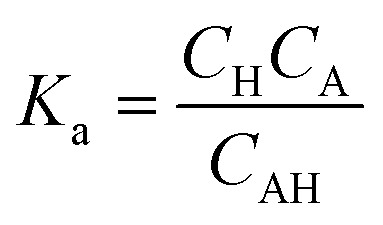
13
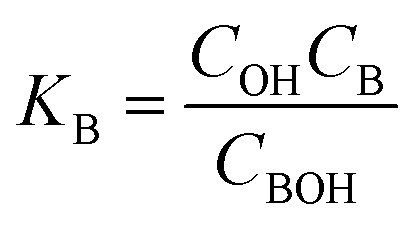
Here, *c*_m_ represents the concentration of the acidic group, acidic conjugate with constant charge, basic conjugate with constant charge, hydroxide, hydrogen ion, and basic groups denoted as AH, B, A, OH, H, and BOH, respectively. In spite of this technique, the total concentration of ionic functionalities in pH-sensitive hydrogels can be determined by summing the concentration of associated ionic groups and constant charges present in the networks. This relationship is expressed by Grimshaw *et al.*, which illustrates the ratio of change in volume of swollen to dry hydrogel. Here, *J* indicates the ratio of volume between the swollen and dry states of a hydrogel.^160^14
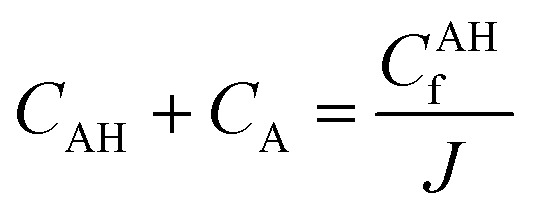
15
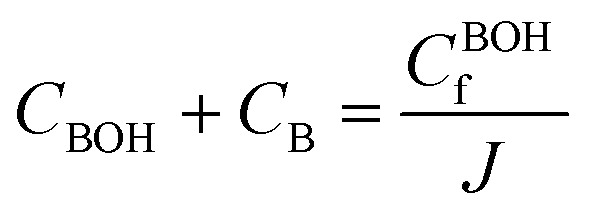


The concentration of constant charges can be obtained by combining eqn (12) and (13) for anionic hydrogels and eqn (14) and (15) for cationic hydrogels,^160^ as follows:16
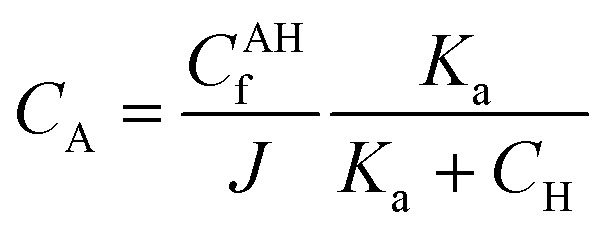
17
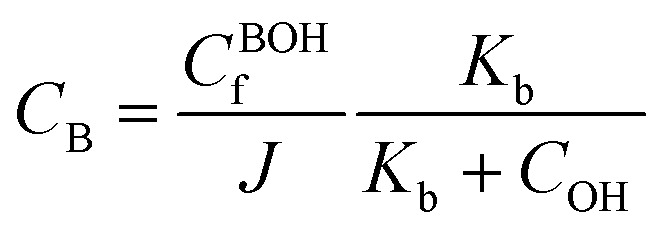


For simplicity, we write anionic hydrogel equations, which are also pH sensitive. However, the movement of ionic species typically arises from diffusion, convection, and electrical migration. The flux is mathematically described by the Nernst–Planck equation, as outlined by Kirby.^161^

18 *j*_*m*_ = *ϕ*(−*D*_*m*_∇_*C*_*m*__ − *z*_*m*_*μ*_*m*_*F*_*C*_*m*__∇*ψ*) + *c*_*m*_*v*

The Debye length is a measure of the EIs between charged particles in the gel. The Debye length describes how far the double layer goes in pH-responsive gels when the pH level changes and there are different ionic atmospheres (higher or lower salt concentrations) in the solution. The variable *f* expresses the hydrogel's porosity, and *D*_*m*_ stands for the *m*th species' diffusion coefficient. The number of charges on ionic species is shown by *z*_*m*_, the mobility of the *m*th species' ions is shown by *μ*_*m*_, *F* stands for Faraday's constant, *c* stands for electric potential, and *v* stands for fluid velocity in relation to the polymer network. The Einstein equation governs the connection between diffusivity and ionic mobility. The relationship between diffusivity and ionic mobility is given by the Einstein equation.

Hydrogels responsive to pH changes, the Nernst–Planck equation often emphasizes the concentration gradient of species when there is no external electric field. Simply put, diffusion takes precedence over convection and electrical migration since OP primarily drives swelling.^162,163^ However, to ascertain the spatial electric field in eqn (18), one can employ Poisson's equation, as elucidated by Jeans.^163^19
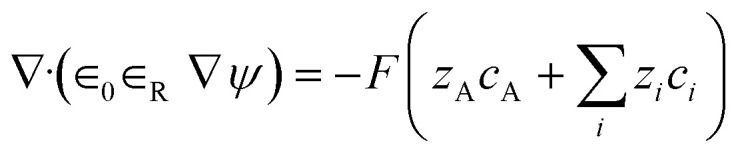


This equation is specifically applicable to anionic gels. It's important to note that in this expression, denotes the permittivity of the vacuum and the gel, respectively. The normalized representation of Poisson's equation takes the following form:20
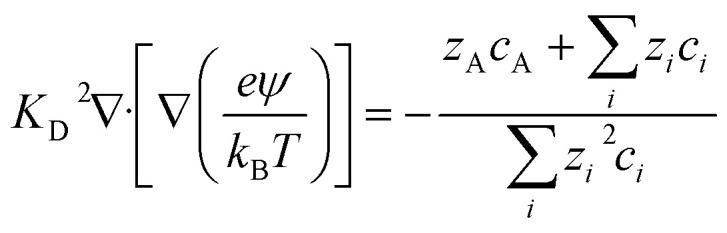
Here, *k*_D_ is defined as the Debye length. The electrostatic charge's influence is characterized by the Debye length, illustrating the extent to which electrostatic effects persist. Beyond the Debye length and at distances surpassing charge-discontinuity regions, including the interior surface, electro-neutrality is balanced both within the gel network and at the gel–solvent interface. The figure depicts that the Debye length typically falls within the nanometre range for polymeric gels.^164^ When the characteristic size of gels exceeds the Debye length, the left-hand side of eqn (13) becomes negligible across the hydrogel volume, excluding the double layer's interior. The double layer, comprising two parallel layers of charge enveloping the hydrogel, is present both externally and internally. The first layer is comprised of ions that have been adsorbed onto the hydrogel, whereas the following one is generated by ions that are drawn to the surface charge as a result of the Coulomb force. Nevertheless, electro-neutrality assumptions warrant revaluation when the hydrogel size approaches the Debye length.^165^

### 4.6. Effect of structural properties on swelling behaviour

The SR of ionic polymers is influenced by several factors, including the inherent mechanical strength of the polymer, the interactions between the polymer and solvent, and the OP resulting from ions present on the interior and exterior surfaces of the gel.^166^ These elements collectively affect the hydrogel's behaviour, which ultimately influences its stability and performance. For instance, in drug delivery applications, the stability and effectiveness of ionic hydrogels can be significantly affected by variations in temperature and pH.^18,167^ When the temperature deviates from a specific range, the gel may undergo phase transitions, leading to alterations in its structure and properties.

Similarly, fluctuations in pH can cause the gel to either expand or contract, impacting the kinetics of drug release.^168^ Thus, designing hydrogels with specific drug delivery characteristics necessitates a thorough understanding and control of these factors.^169^ For example, changes in pH can be utilized to trigger drug release in pH-responsive hydrogels, enhancing the controlled delivery of therapeutics.^170,171^ The differential diffusion of drug molecules across gels that swell or shrink in response to pH variations allows for improved therapeutic effectiveness and enables the development of customized treatment strategies.

OP and electrostatic repulsion within polyelectrolyte gels are key factors influencing hydrogel swelling behaviour, particularly in quaternized chitosan/carrageenan hydrogels. The swelling capacity depends on the gel's chemical composition. In cationic polyelectrolyte gels, repulsive forces between fixed positive charges push water molecules and polymer chains apart, leading to expansion. Conversely, in anionic polyelectrolyte gels, fixed negative charges attract water molecules, promoting swelling and water absorption. These distinct swelling mechanisms highlight the importance of optimizing hydrogel properties through a deeper understanding of their chemical composition and interactions. Interestingly, cationic polyelectrolyte gels can exhibit water absorption without the expected repulsion between water molecules and polyelectrolyte chains. In some cases, favorable interactions between fixed positive charges and water molecules promote hydration, challenging the assumption that positive charges always induce repulsion. This highlights the need for a detailed understanding of chemical composition and interaction mechanisms in material design. Researchers have successfully engineered positively charged hydrogels with high water absorption capacity, making them ideal for applications requiring high water content, such as contact lenses and wound dressings.

Furthermore, researchers have developed nanomaterials with unique surfaces that exhibit both hydrophilic and hydrophobic characteristics.^172,173^ These materials have proven beneficial in oil spill cleanup applications, selectively attracting and absorbing organic molecules while repelling water. This advancement refutes the idea that materials can possess only a singular characteristic and emphasizes the need for a comprehensive understanding of chemical compositions and interactions to create multifunctional materials. For instance, this nanomaterial can effectively separate oil droplets from water due to its hydrophobic surface, which prevents water from mixing with the oil.^174^ At the same time, the hydrophilic surface draws in and absorbs organic contaminants, facilitating efficient spill remediation. This innovative approach demonstrates how materials with dual functionalities can revolutionize environmental cleanup efforts.

Another notable example is the development of self-healing materials that leverage adaptable components with specific functions. These materials can autonomously repair themselves when damaged, reducing the need for regular maintenance and replacement. For instance, a self-healing polymer coating on a vehicle's exterior can repair minor dings and scratches over time, thereby preserving the vehicle's integrity and aesthetic appeal.^175^ This breakthrough in material science has the potential to significantly enhance durability and reduce waste across various industries. Additionally, advances in regenerative medicine may lead to the creation of medically functional, self-healing tissues and organs. Researchers are exploring methods, such as utilizing stem cells, to repair damaged heart tissue following myocardial infarction. Such innovations could potentially eliminate the need for heart transplants in patients with heart disease, significantly improving their quality of life.

### 4.7. Effect of stimuli on swelling behaviour

Stimuli-sensitive hydrogels can undergo significant changes in shape or volume in response to specific environmental triggers.^176^ These stimuli can include physical factors such as light, pressure, temperature, electric and magnetic fields, and ultrasound. Additionally, they can respond to chemical stimuli, including glucose, CO_2_, ionic strength, pH, and redox conditions.^177,178^ Biological signals, such as DNA, glutathione, antigens, and enzymes, may also activate these hydrogels.^179,180^ Stimuli influencing hydrogels can be classified as internal or external, based on their *in vivo* origin. Chemical and biological factors are internal stimuli, while physical factors (excluding temperature) are external stimuli. Often termed “smart” or “intelligent” hydrogels, these materials can detect stimuli and respond by modifying their physical or chemical properties, enabling the controlled release of encapsulated drugs.

Among stimuli-responsive polymeric gels, pH-sensitive hydrogels have attracted significant research interest due to their ability to undergo abrupt volume phase transitions, resulting in swelling or collapse under specific conditions. Their responsiveness depends on factors such as size, shape, cross-linking density, ionic group content, and overall composition, all of which can be tailored accordingly. The response rate varies based on pore size, ionic group concentration, and cross-linking density. Another intriguing class of intelligent materials is shape-memory polymeric hydrogels, which can retain a fixed shape and recover it upon exposure to a specific trigger. Achieving shape memory requires (i) a polymer that undergoes phase transitions in response to stimuli and (ii) a memory code (chemical or physical) that enables shape recovery when triggered. Guo *et al.*,^181^ designed pH-sensitive hydrogels capable of shape memory using acrylamide and deoxyribonucleic acid (DNA) as cross-linked polymers. At pH 5, the hydrogel maintains its shape due to cross-linked polyacrylamide and duplex bridges within the DNA. Conversely, at pH 8, the hydrogel deforms and enters a quasi-liquid state, where the DNA duplex bridges do not contribute to restoring the hydrogel to its original shape upon returning to pH 5.

Redox-responsive hydrogels are a unique class of intelligent materials that react to reducing conditions, particularly elevated levels of glutathione (GSH) tripeptide. These hydrogels incorporate strategically placed disulfide linkages within their main or side chains or as cross-linkers. In the circulatory system and extracellular matrix (ECM), where glutathione levels are low (2.0–20 μM), disulfide bonds remain stable. However, inside cells, where glutathione concentrations rise to 0.5–10 mM, these bonds undergo thiol-disulfide exchange, leading to rapid cleavage. Notably, tumour tissues have glutathione levels four times higher than normal tissues, causing disulfide bond disruption and facilitating drug or bioactive compound release from the intracellular matrix. Yu *et al.*,^182^ synthesized hydrogels that can be reduced by incorporating poly(ethylene glycol) monomethyl ether (mPEG). This compound, mPEG-g-SS-PAA, consists of disulfide-linked poly(amido-amine) grafted with α-cyclodextrin. The researchers integrated BSA into the hydrogels and investigated how reductions in the intracellular matrix affected its release.

**Fig. 11:**
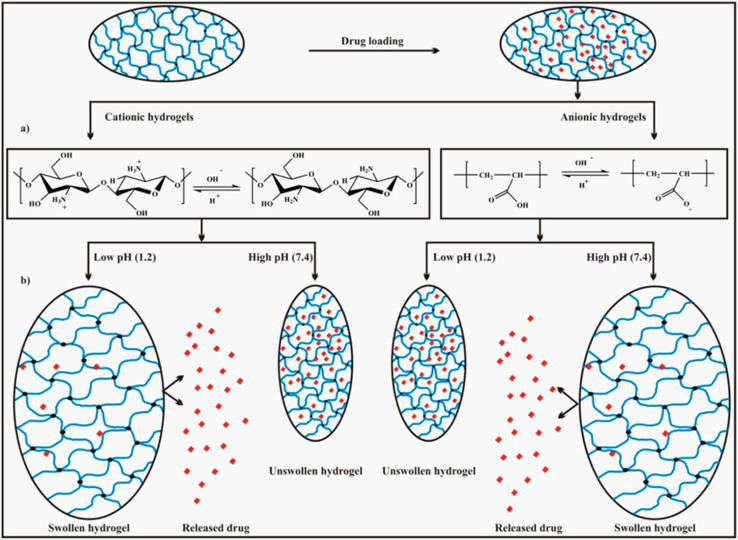
(a) pH-dependent ionization of specific acidic or basic functional groups on hydrogel chains responsible for swelling, (b) pH-dependent swelling and drug release mechanism. The figure is adapted from ref. 184 Copyright 2017, MDPI.

Ionic hydrogels exhibit pH-responsive swelling due to the presence of pendant groups with charges.^183^ Several variables influence this swelling behaviour, including ionic charge, the pKa or pKb values of ionizable groups, degree of ionization, hydrophilicity, polymer content, and the pH of the swelling medium. The composition of pendant groups and the pH of the environment are critical determinants of the properties of pH-sensitive hydrogels. For instance, cationic hydrogels such as poly(ethylene imine) and chitosan expand in acidic environments as amino/imine groups become protonated, leading to the formation of positively charged species that induce repulsion and swelling as shown in Fig. 11.^184^ These hydrogels are advantageous for drug delivery in the stomach within injectable drug delivery systems. In contrast, anionic hydrogels such as carboxymethyl chitosan swell in alkaline solutions due to the ionization of their acidic groups. The presence of negatively charged pendant groups induces repulsion and swelling, making these hydrogels suitable for drug administration in the gut at a physiological pH of 7.4. Another effective drug delivery strategy involves using polyelectrolyte complex (PEC) hydrogels, which eliminate the need for hazardous covalent cross-linkers. PEC hydrogels typically consist of a positively charged polymer, like chitosan, and a negatively charged polymer, such as carboxymethyl chitosan, held together by Eis. Zaino *et al.,*^185^ studied the drug delivery characteristics of dexamethasone using pH-sensitive PEC hydrogels composed of *N*-trimethyl chitosan (positively charged) and *N*-carboxymethyl chitosan (negatively charged).

The swelling behaviour of hydrogels upon exposure to water or physiological fluids is primarily driven by OP, influenced by the hydrophilic nature of polymers, static charges within the polymer network, and counterions present in the hydrogel matrix. This process occurs in three sequential stages: (i) initial water infiltration into the hydrogel network, (ii) polymer chain relaxation and hydration, and (iii) expansion of the network as polymer chains continue to relax. Swelling is primarily induced by primary-bound water, which forms through interactions between hydrophilic and polar groups and water molecules. Simultaneously, hydrophobic regions interact with water, leading to the formation of secondary-bound water. The overall increase in water content is regulated by osmotic forces, counteracted by the elastic retractive forces of cross-linked polymer chains. As the swelling progresses, excess water (free water) accumulates, guiding the hydrogel toward equilibrium swelling. At this point, swelling is stabilized by a delicate balance between elastic retractive forces and OP. This phenomenon is well explained by the Flory–Rehner theory, which attributes hydrogel swelling to the elastic properties of polymer chains and the thermodynamic compatibility between water molecules and the polymer matrix.^186,187^ A volume-phase transition occurs when stimulus-responsive hydrogels interact with specific triggers. The swelling behaviour of ionic hydrogels is primarily governed by two factors: (i) the intrinsic properties of the hydrogel-forming polymer, including cross-linking density, hydrophilicity, ionic charge, p*K*_a_, and p*K*_b_ values; and (ii) the characteristics of the swelling medium, including ionic concentration, acidity, and counterion types.

The rate of water uptake in hydrogels is contingent upon the surrounding medium's pH in relation to the p*K*_a_ and p*K*_b_ values of the pendant groups in the polymer chains.^188^ For an anionic network (*e.g.*, with carboxylic acid, COOH pendant groups), when the surrounding medium's pH exceeds the p*K*_a_ of the acidic pendant groups, ionization occurs, resulting in fixed negative charges on the polymer chains and mobile positive charges in the solution. This change affects (i) the hydrophilicity of the hydrogels, (ii) the quantity of fixed negative charges, and (iii) the electrostatic repulsion between the chains, leading to an expansion of the hydrogel network; the reverse occurs when pH is lower than the p*K*_a_. Conversely, in a cationic network (*e.g.*, containing amino or NH_2_ pendant groups), if the pH of the surrounding medium is below the p*K*_b_ of the pendant basic groups, protonation occurs, increasing the number of fixed positive charges and inducing swelling due to enhanced hydrophilicity and electrostatic repulsion. Conversely, swelling contracts when the pH exceeds the p*K*_b_.

Zare-Akbari *et al.*,^189^ synthesized ionic cross-linked hydrogel beads based on CMC and ZnO nanoparticles (NPs) using Fe^3+^ ions as a physical cross-linking agent. The prepared hydrogel beads exhibited pH-sensitive swelling behaviour when immersed in phosphate-buffered saline (PBS) at pH levels of 1.2, 6.8, and 7.4 at room temperature, simulating various physiological environments. The swelling degree at pH 7.4 and 6.8 was found to be approximately three times greater than that at pH 1.2. At pH 6.8 and 7.4, the carboxyl groups in CMC dissociated in aqueous media, converting to negatively charged carboxylate ions, which led to increased electrostatic repulsion and enhanced water penetration into the hydrogel matrix. The pH-sensitive drug release behaviour was demonstrated using propranolol hydrochloride as a model drug, suggesting that the hydrogel beads could be potential candidates for controlled drug delivery applications.

Additionally, various polymers containing weakly acidic or basic groups that readily undergo hydrolysis or protonation have been developed for pH-sensitive hydrogels with cellulose, including gelatine, PAA, hyaluronic acid (HA), and poly(*N*,*N*-dimethylaminoethyl methacrylate) (PDMAEMA). The swelling behaviour of these hydrogels can be modulated by altering the pH, enabling controlled release of therapeutic agents.^190^ Furthermore, hydrogels with anionic and cationic polymeric networks can be synthesized through polyelectrolyte complexation, where cationic and anionic polymers form stable complexes, leading to enhanced swelling capacity in response to changes in pH.

## 5. Applications of cellulose-based hydrogels

In the biomedical field, hydrogels are prominent among polymer types due to their unique properties that render them suitable for various applications. These materials exhibit characteristics such as compatibility with living tissue, which opens numerous possibilities for use in biomedical settings. Their resemblance to biological tissues creates significant opportunities in areas such as drug delivery, wound care, and the rapidly advancing field of tissue engineering. The diverse attributes of hydrogels make them invaluable, offering versatile solutions to complex biomedical challenges. However, while different polymer types may possess unique properties for biomedical applications, their similarity to living tissue does not necessarily guarantee efficacy in drug delivery, wound care, or tissue engineering.

### 5.1. Drug delivery

Hydrogel-based drug delivery systems demonstrate efficacy in precisely targeting and delivering drugs to specific sites within the body.^191–193^ These hydrogels can respond to various environmental stimuli, including light, temperature, pH fluctuations, and chemical interactions, as well as electric and magnetic fields.^22^ Their intriguing ability to expand and contract facilitates the clustering of polymers, leading to changes in transparency, size, and the formation of hydrogen bonds between molecules. Upon disruption of the hydrogel structure, drugs encapsulated within are released, with swelling occurring in response to nearby stimuli. Understanding the swelling process is crucial for studying the composition of hydrogels and its impact on response rates. Moreover, the degree of swelling influences the mechanical properties of the hydrogel, including flexibility and durability.^194^ A deep understanding of swelling behaviour is crucial for optimizing hydrogels in drug delivery and tissue engineering. Their stimuli-responsive nature enables precise and controlled drug release, improving therapeutic outcomes. The incorporation of cellulose and its derivatives significantly modifies hydrogel structures, creating larger pores due to intra-carboxyl group repulsion, which enhances the SR. Additionally, cellulose's responsive properties make it an excellent candidate for regulated drug release. Its proven biocompatibility, as confirmed by viability assessments, further supports its potential for biomedical applications. Numerous studies have focused on the role of hydrogels in regulating drug release. For instance, Villalba-Rodríguez *et al.*,^195^ conducted a comprehensive review on drug delivery using biodegradable hydrogels made from various materials, including chitosan, poly (lactic-*co*-glycolic acid), and bacterial cellulose. Hydrogels that adapt to changes in temperature, pH, and redox conditions exhibit considerable potential for drug delivery, aligning well with the body's natural mechanisms. Researchers have developed membranes from pH-responsive CMC-based hydrogels for applications in drug release and wound care. Distinct volumetric characteristics of HPC and poly(*N*-isopropyl acrylamide) (PNIPA) hydrogels have also been highlighted, indicating their promise in diverse biomedical applications.^196^ Additionally, thiolated HPC has shown potential as a hydrogel responsive to both redox reactions and temperature variations, making it suitable for controlled drug release.

**Fig. 12:**
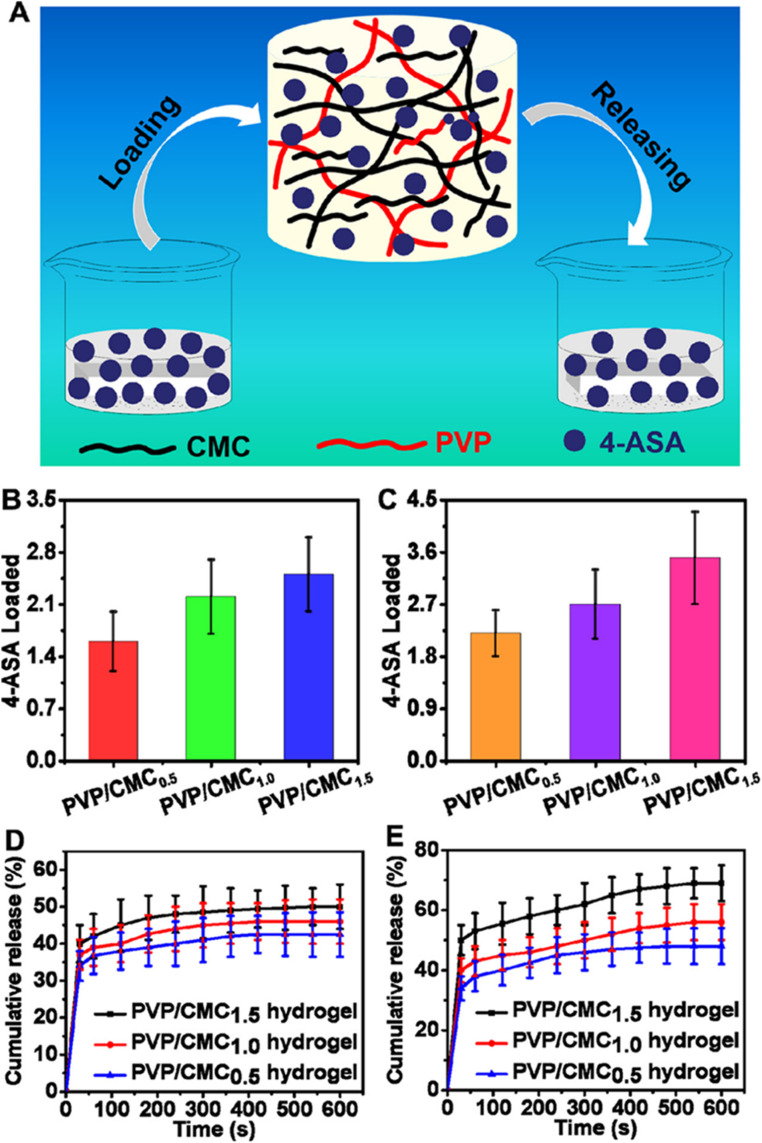
(A) A diagram illustrating the drug loading and release process of the PVP/CMC hydrogel. The loading behaviour of the hydrogel at varying CMC concentrations in PBS is shown in (B) at pH 2.0 and (C) at pH 7.4. The release profile of 4-ASA from different hydrogel formulations over time in PBS is depicted in (D) at pH 2.0 and (E) at pH 7.4. The figure is reprinted from ref. 197. Copyright © 2024 The Authors. Published by American Chemical Society. This publication is licensed under CC-BY-NC-ND 4.0.

Zuwu Tang *et al.*^197^ developed a biocompatible, injectable, and self-healing PVP/CMC hydrogel using *N*,*N*′-methylenebis(acrylamide) (MBA) as a crosslinker and potassium persulfate (KPS) as an initiator. This hydrogel was designed for drug encapsulation and controlled release of 4-ASA, a drug commonly used to treat tuberculosis and inflammatory bowel diseases. Given that 4-ASA is sensitive to heat and moisture, its amphoteric nature-which depends on the surrounding pH-made it a suitable water-soluble model drug for this study. The drug loading and release properties of PVP/CMC hydrogels were tested in PBS buffer solutions at pH 2.0 and 7.4 at 37 °C (Fig. 12A). Increasing CMC concentration led to higher drug loading due to the greater availability of carboxyl and hydroxyl groups, which interacted with 4-ASA *via* hydrogen bonding. At 1.0 g CMC content, drug loading was 2.5 in pH 2.0 and 3.5 in pH 7.4, with higher loading at pH 7.4 due to reduced hydrogen bond interference in a less acidic environment. Drug release profiles (Fig. 12D and E) showed that hydrogels at pH 7.4 released more drug than those at pH 2.0. For instance, the PVP/CMC1.5 hydrogel released 70% of the drug in pH 7.4, compared to only 50% in pH 2.0. This difference was due to protonation of CMC's carboxyl groups at low pH, which led to hydrophobic region formation, causing the hydrogel network to contract and restrict drug diffusion. Overall, this study demonstrated that CMC concentration and pH significantly influence the drug loading and release behaviour of PVP/CMC hydrogels, making them promising for controlled drug delivery applications. In contrast, at pH = 7.4, the electrostatic repulsion among ions weakens the contraction, facilitating drug release.^197^ Additionally, as the CMC content increased, the drug release rate also increased. These findings suggest that the PVP/CMC hydrogel is highly efficient for drug release, and both the loading and release of 4-ASA are pH-dependent and can be optimized by adjusting the composition of the PVP/CMC hydrogel.^197^

Many studies provide insights into the release profiles of various proteins using a range of hydrogel types, including dextran, ketoprofen, BSA, and alaptide.^198^ Various hydrogels utilized in these studies encompass interpenetrating networks (IPN), semi-IPN HPC-poly(acrylic acid), polyacrylamide grafted with xanthan gum (PAAm-g-XG), and different cellulose variations. Dutta *et al.*,^199^ reported on the development of triple stimuli-responsive hydrogels demonstrating the versatility of CMC and PNIPA. Research continues to explore various types of cellulose hydrogels that selectively release drugs in response to stimuli, including pH, magnetic fields, and temperature.^200^ While cellulose hydrogels function optimally in neutral environments, chitosan-based hydrogels are particularly effective in acidic conditions, which corresponds with the diverse pH levels found in different organs.

Fekete *et al.*,^201^ discussed how CMC/starch superabsorbent hydrogels synthesized *via* gamma irradiation exhibit superior water absorption compared to pure cellulose hydrogels, especially at high electrolyte concentrations. Conversely, cellulose/pectin hydrogels have been designed for targeted delivery to the colon, relying on their adhesive properties and enzymatic degradation.^202^ pH-sensitive hydrogels are emerging as prominent carriers for oral drug delivery to the intestine or colon, demonstrating their potential in non-invasive drug delivery strategies. Ahmadi *et al.*,^203^ highlighted the versatility of chitosan-based hydrogels for drug delivery systems *via* multiple routes, including oral, ocular, and nasal administration. In a different context, Vlaia *et al.*,^204^ emphasized the role of cellulose-derived hydrogels in dermal and transdermal drug delivery systems, showcasing the flexibility of cellulose hydrogels for diverse drug delivery needs. While these examples illustrate the potential of hydrogels in drug delivery, they do not confirm widespread clinical application or effectiveness.

Beyond drug delivery, cellulose-based hydrogels present substantial opportunities for advancements in disease detection and treatment.^205^ Various NPs, including lipid-based celluloses, liposomes, polymeric celluloses, dendrimers, carbon nanomaterials, and both inorganic and metallic NPs, are integrated with cellulose derivatives.^206^ This incorporation enhances the solubility and bioavailability of active pharmaceutical compounds, which is vital for developing dosage forms and achieving accurate release profiles in pharmaceutical formulations. However, concerns about the potential risks and long-term effects of NPs in drug delivery systems must be addressed, as enhanced drug absorption and solubility may not justify these hazards. Additionally, the use of NPs may increase production costs and limit the accessibility of certain medications.^207,208^

In cancer treatment, NPs can be employed to reduce the adverse effects on healthy tissues by selectively delivering chemotherapeutic agents to tumour cells.^209^ This targeted drug delivery approach enhances therapeutic efficacy while minimizing toxicity. However, concerns about the potential long-term effects and health risks associated with nanoparticle accumulation in organs and tissues persist.^210^ Moreover, the complex manufacturing processes and specialized equipment required for integrating NPs into drug formulations can significantly escalate production costs, restricting access to these therapies. Researchers are actively developing nanoparticle-based drug delivery systems in oncology to enhance targeting and reduce harm to healthy organs while effectively treating cancer cellulose.

Farnoush Ahmadpour *et al.*,^211^ conducted research exploring the distinctive characteristics and multifunctionality of hydrogel structures used in biomedicine. In their study, they synthesized a cross-linked Pec-Cel hydrogel using CaCl_2_ as a cross-linker. The synthesis of Fe_3_O_4_ magnetic NPs (MNPs) was then performed in the presence of the cross-linked hydrogel, resulting in the formation of a novel magnetic cross-linked Pec-Cel hydrogel nanobiocomposite. To assess the efficacy of this magnetic nanobiocomposite for *in vitro* hyperthermia applications, varying concentrations of the nanostructure were tested under alternating magnetic fields (AMF) with different frequencies. As anticipated, the highest specific absorption rate (SAR) was observed in the sample with the lowest concentration of magnetic nanobiocomposite, as the dipole–dipole interactions between NPs can disrupt their heat generation. Moreover, a significant reduction in SAR was noted as the sample concentration increased from 0.5 mg mL^−1^ to 10.0 mg mL^−1^, with SAR dropping from 126.0 W g^−1^ at 0.5 mg mL^−1^ to approximately 5.0 W g^−1^ at 10.0 mg mL^−1^. While the sample concentration had a substantial impact on the SAR, the influence of AMF frequency was comparatively less pronounced.^211^ The SAR decreased by over 95.0% as the concentration increased from the lowest to the highest, while the variation in SAR due to changes in AMF frequency was only 6, 7, 14, 30, and 2 percent for the 0.5, 1.0, 2.0, 5.0, and 10.0 mg mL^−1^ samples, respectively. These results indicate that the effect of AMF frequency differs based on sample concentration. Although the data shows that lowering the concentration of the magnetic Nanobiocomposite increases the thermal power per unit mass, it is crucial to also compare the temperature differences generated by each sample for a more comprehensive evaluation of their performance.^211^

### 5.2. Wound healing

The natural healing of wounds involves a complex interplay of biological systems that work together to restore the integrity of damaged skin.^212^ During this intricate process, tissue layers and cellulose Lular structures are replaced. For example, Hao *et al.* developed an injectable gel that aids in the healing of diabetic wounds by preventing infections and promoting angiogenesis.^213^ Hydrogels have proven effective for treating wounds in both diabetic and healthy individuals.^11,214^ Cellulose hydrogels and their derivatives, available in various forms such as fibres, membranes, and sponges, are widely utilized in wound care products.^99,215^ Due to the natural polycationic and hemostatic properties of deacetylated chitin or chitosan, these materials are particularly beneficial for wound healing applications. It is common practice to incorporate zinc oxide or silver NPs into cellulose gel systems to impart antimicrobial effects, as cellulose lacks inherent antibacterial activity.

Initially, hydrogel wound dressings aimed to maintain a moist environment and provide physical protection.^216^ However, as clinical demands for improved performance in wound healing have evolved, hydrogel dressings with enhanced single or multiple biological activities have emerged. In addition to facilitating *in situ* creation and rapid hemostasis, hydrogels serve as essential physical barriers, preventing external bacterial infections.^217^ Nevertheless, conventional hydrogels are susceptible to rupture and damage upon exposure to tissue movement or external strain, compromising their integrity and function while increasing the risk of bacterial invasion and wound infection. Consequently, hydrogel dressings must maintain structural integrity throughout the healing process. Self-healing hydrogels have emerged as an innovative approach characterized by materials that can autonomously repair structural and functional damage.^218^ This technique employs constitutional dynamic chemistry to create a cross-linked hydrogel network through dynamic and reversible chemical interactions. Self-healing hydrogels can be categorized into two types based on their healing mechanisms: physical and chemical.^219^ Physical self-healing hydrogels reconstruct networks through non-covalent interactions, such as hydrophobic interactions, host–guest interactions, hydrogen bonding, crystallization, and various intermolecular forces among molecules or polymer chains. For instance, Liu *et al.*,^220^ developed a composite double-network hydrogel using tannic acid and gelatin methacrylate, with the hydrogel's efficient self-healing properties attributed to the dynamic hydrogen bonds of tannic acid. Another strategy involved modifying bacterial cellulose with both positive and negative fragments to create a self-healing hydrogel in a pH 7.4 buffer solution *via* an ionic interlocking mechanism.

Hao Chen *et al.*,^221^ introduced an injectable, self-repairing hydrogel with antibacterial and angiogenesis-promoting features for the treatment of diabetic wounds. This hydrogel, named Ag-SH-PEG, was synthesized through a straightforward method involving the coordination-based crosslinking of multi-arm thiolated polyethylene glycol (SH-PEG) with silver nitrate. Thanks to the reversible and dynamic nature of the Ag–S coordination bonds, the hydrogel demonstrated both self-healing behaviour after mechanical disruption and the ability to be administered *via* a medical syringe. The researchers proposed that this multifunctional hydrogel could effectively combat infections, promote blood vessel formation, and expedite tissue recovery in diabetic skin wounds.^221^

Additionally, the hydrogel matrix gradually released antimicrobial silver ions, making it particularly suitable for managing vulnerable open wounds in diabetic patients. By incorporating desferrioxamine (DFO), an angiogenic agent, into the hydrogel, the team developed a material that combined stress resistance, antibacterial efficacy, and enhanced angiogenic potential. In their experimental model using full-thickness dorsal skin wounds in SD rats, three groups were treated: a control group, a group receiving the plain hydrogel, and a group treated with the DFO-loaded hydrogel.^221^ Wound healing was assessed on days 0, 4, 7, 10, and 14 through digital imaging and quantitative analysis of wound size using ImageJ software.^221^ By day 7, wounds treated with the hydrogel showed significantly smaller diameters, with those in the DFO-hydrogel group shrinking by 50% of their original size. This reduction correlated with better tissue repair and a dry appearance, suggesting prevention of bacterial contamination and early tissue remodeling. In contrast, wounds in the control and plain hydrogel groups showed only a 30% size reduction by the same time. By day 14, nearly complete closure was observed in the DFO-hydrogel-treated wounds.^221^ The comparative analysis of wound areas revealed minimal differences between the groups during the initial inflammatory phase (first 2–3 days post-injection), likely due to limited vessel formation and epithelialization during this period. However, a noticeable acceleration in wound closure occurred after day 7 in the DFO-hydrogel group, persisting until day 14. The marked difference in wound size between this group and the others highlighted the biocompatibility of the DFO-loaded hydrogel and its ability to support tissue regeneration effectively.^221^

Esteban Guamba *et al.*,^222^ extracted cellulose from four distinct plants native to Ecuador and utilized it as a polymer to synthesize cellulose-based hydrogels. These hydrogels were subsequently compared to a reference hydrogel derived from commercially available cellulose. Comprehensive characterization was performed on both the cellulose powder isolates and the resulting hydrogels. Notably, cellulose from pear mesocarp (F1) failed to produce a hydrogel, leaving hydrogels prepared from pear epicarp (F4), tomato (F12), pitahaya (F53), and commercial cellulose for further investigation. These selected hydrogels underwent *in vitro* antimicrobial assays and were evaluated for wound dressing applications using a pigskin model as a conceptual demonstration.^222^

The findings revealed that hydrogels fabricated using pitahaya cellulose demonstrated the highest potential in inhibiting bacterial proliferation, making them favourable for wound healing applications.^222^ This efficacy was attributed to their antifouling properties, which appeared to impede bacterial adhesion and growth. However, all hydrogels-except for F53-exhibited enhanced bacterial colonization, with the commercial wound dressing showing the most significant colony formation under identical conditions. Interestingly, F53 effectively controlled bacterial growth within the initial 48 hours, after which normal bacterial proliferation resumed. These observations highlight F53 as the most promising candidate for developing wound dressings, surpassing both commercial cellulose-based hydrogels and standard wound bandages in bacterial growth resistance. While hydrogels from F12, CMC, and F53 displayed no colony formation in the *in vitro* tests, only F53 demonstrated reduced bacterial growth in the *ex vivo* pigskin model. This discrepancy is likely due to the variation in bacterial strains present in pigskin samples compared to the single-strain inoculum used in the *in vitro* assessments.^222^

**Fig. 13:**
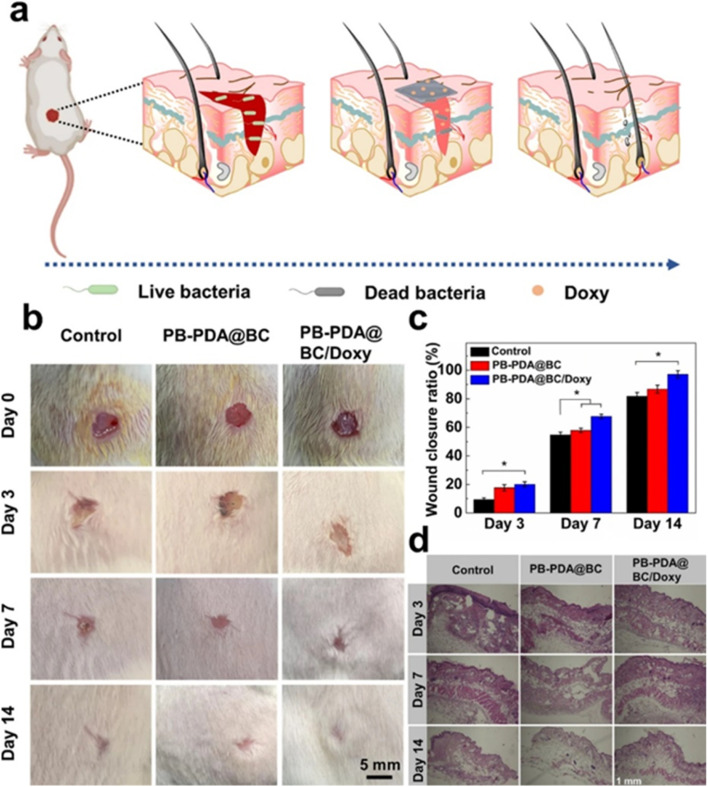
Highlights the application and effectiveness of hydrogels in wound healing. Panel (a) demonstrates the use of the hydrogel as a therapeutic agent to enhance the wound recovery process. Panel (b) compares the wound closure rates achieved through various treatments, showcasing the superior efficacy of the PB-PDA@BC/Doxy hydrogel. Panel (c) provides histological evidence of wound regeneration on the 3rd, 7th, and 14th days, comparing commercial Tegaderm film dressing, PB-PDA@BC, and PB-PDA@BC/Doxy hydrogels. Notably, the PB-PDA@BC/Doxy hydrogel exhibits enhanced regenerative capabilities, as evidenced by improved tissue organization and closure over time. Finally, panel (d) offers a detailed depiction of the PB-PDA@BC/Doxy hydrogel actively promoting wound healing, underlining its potential as an advanced material in tissue repair applications. The figure is adopted from ref. 223. Copyright © 2023, The Author(s), under exclusive licence to Springer Nature B.V.

Xiaotong Yi *et al.*,^223^ designed and developed an innovative, adhesive, antibacterial, self-healing hydrogel wound dressing by incorporating a PVA-borax hydrogel matrix with bacterial cellulose (BC), dopamine (DA), and doxycycline (Doxy). To improve the adhesion and mechanical strength of the PB hydrogel, a reinforcement material, PDA@BC, was synthesized *via* dopamine-induced self-polymerization. This material was then coated onto the BC surface, allowing for uniform dispersion within the hydrogel matrix. The resulting PB-PDA@BC hydrogels, loaded with doxycycline, exhibited outstanding antibacterial properties. These effects were primarily due to the local release of the antibiotic, alongside the hydrogels' favourable cytocompatibility and blood compatibility.^223^ The wound healing efficiency of the PB-PDA@BC/Doxy hydrogel was compared to the commercial Tegaderm film dressing (control group) across three time points-day 3, day 7, and day 14-as shown in Fig. 13(a–c). All groups demonstrated a reduction in wound area, but the PB-PDA@BC/Doxy group exhibited the most significant wound closure, with a 20.1% reduction on day 3 (*p* < 0.05). By day 7, the PB-PDA@BC/Doxy hydrogel group showed a 5.7% and 11.9% improvement in wound closure compared to the PB-PDA@BC hydrogel and control groups, respectively. On day 14, the PB-PDA@BC/Doxy group achieved near-complete healing, with a wound closure of 97.1%, which was approximately 10% higher than the control group, as confirmed by quantitative analysis of the wound area (*p* < 0.05). These results suggest that doxycycline-loaded PB-PDA@BC hydrogels offer superior performance in wound healing compared to Tegaderm, thanks to the combined effects of antibiotic properties, hemostasis, and the moist environment provided by the hydrogel matrix. However, the antibiotic-coated PB-PDA@BC hydrogel efficiently eliminates bacteria at the wound site, promotes a conducive microenvironment for cellular proliferation and epidermal regeneration, and accelerates the overall healing process. To further assess the wound healing process, tissue samples from the wound area were subjected to histological analysis.^223^ Hematoxylin and eosin (H&E) staining results (Fig. 13(d)) revealed mild inflammatory responses across all groups after 3 days of treatment. However, the PB-PDA@BC/Doxy hydrogel group showed significantly fewer inflammatory cells compared to the control group, likely due to the antibacterial effects of doxycycline. After 7 days, all groups exhibited the formation of a new epidermal layer. Notably, the PB-PDA@BC/Doxy hydrogel group displayed a thicker epidermis than both the control and PB-PDA@BC hydrogel groups. By day 14, the PB-PDA@BC/Doxy group also showed the emergence of hair follicles and blood vessels, signalling the hydrogel's ability to support skin regeneration. These findings demonstrate that the drug-loaded hydrogels not only enhance wound healing but also promote skin regeneration, making them a promising option for accelerated tissue repair. However, challenges remain in developing hydrogels that provide the optimal balance of structural integrity, mechanical flexibility, and controlled drug release for effective wound healing. Hydrogels with superior mechanical properties can withstand various stresses while providing sufficient softness and pliability for enhanced comfort in wearability. Developing next-generation cellulose hydrogels that address these factors will significantly improve wound care solutions.^223^

### 5.3. Tissue engineering

Tissue engineering requires hydrogels that fulfil specific criteria, balancing physical properties such as degradation rates and mechanical strength with biological performance indicators like cell adhesion.^224,225^ Biocompatibility is of paramount importance, ensuring that these hydrogels can exist within the body without causing harm or eliciting adverse responses.^226,227^ This aspect is particularly crucial, as any inflammatory reaction to the hydrogel could influence the immune response towards transplanted cells and *vice versa*. Naturally derived polymers generally exhibit suitable biocompatibility; however, synthetic polymers may trigger negative bodily reactions, which limits their application in hydrogel preparation for tissue engineering purposes.^228^

A promising strategy for treating patients in need of new organs or tissues involves the engineering of artificial tissues or organs.^229^ Various techniques have been developed for this purpose, with one particularly appealing approach involving the use of a combination of patient-derived cells and polymer scaffolds.^230,231^ Cells isolated from a patient's tissue biopsy are cultured *in vitro* and subsequently integrated into three-dimensional polymer scaffolds that mimic the natural extracellular matrices found in tissues. These scaffolds facilitate cell delivery to the targeted site, create space for new tissue formation, and may regulate the structure and function of the engineered tissue. This approach has been employed in engineering different types of tissues, including arteries, bladders, skin, cartilage, bone, ligaments, and tendons, some of which are nearing clinical use. Additionally, techniques have been developed to guide cells towards the desired phenotype, a critical aspect of successful tissue engineering.

CNCs, typically isolated from cellulose, have gained popularity as reinforcing fillers in polymeric hydrogels due to their favourable mechanical properties and intrinsic biocompatibility.^232,233^ The unique aspect ratio and abundant active –OH groups on their surface make CNCs an excellent precursor for preparing robust stabilizers. Yang *et al.*,^234^ reported injectable hydrogels based on adipic acid dihydrazide-modified CMC and aldehyde-modified dextran, reinforced with CNCs and aldehyde-functionalized CNCs. Gelation occurred within seconds when the hydrogel components were extruded from a double-barrel syringe, and CNCs were observed to be evenly distributed throughout the composites. Swelling tests indicated that all CNC-reinforced hydrogels maintained their structural integrity for over 60 days in both water and 10 mM PBS, suggesting their potential for long-term applications. The CHO-CNCs acted as both a filler and a chemical cross-linker, enhancing the elasticity and stability of the CHO-CNC-reinforced hydrogels, allowing for higher nanoparticle loadings without compromising mechanical strength compared to hydrogels containing unmodified CNCs. Cytotoxicity tests demonstrated that both the initial components and the hydrogels exhibited good cytocompatibility with NIH 3T3 fibroblast Cells. Together with their syringe ability, these CNC-reinforced injectable hydrogels show promise for filling irregular cavities and shapes without requiring pre-shaped forming processes, making them ideal for bone tissue engineering.^234^

The introduction of inorganic NPs, such as LAPONITE^®^, hydroxyapatite (HAp), and titanium dioxide, can further enhance cellulose-based hydrogels for bone tissue engineering applications.^235^ Boyer and colleagues,^236^ developed a LAPONITE^®^ NP-reinforced Si-HPMC hydrogel, in which LAPONITE^®^ NPs self-assembled within the Si-HPMC gel structure, resulting in a hybrid interpenetrating network (IPN). This IPN structure significantly improved the mechanical properties of the hydrogel while maintaining oxygen diffusion and cell viability after gelation. The ability of the hybrid scaffold, composed of the Si-HPMC/LAPONITE^®^ hydrogel and chondrogenic cells, to form cartilaginous tissue *in vivo* was investigated over six weeks of implantation in subcutaneous pockets of nude mice. Histological analysis of the composite constructs revealed the formation of cartilage-like tissue with an extracellular matrix rich in glycosaminoglycans and collagen. These findings indicate that the prepared Si-HPMC/LAPONITE^®^ hydrogels possess considerable potential for repairing cartilage defects. Furthermore, Tohamy *et al.*,^237^ constructed composite scaffolds of sodium alginate (SA)/HEC/HAp using a lyophilization technique followed by cross-linking in the presence of Ca^2+^ ions. The study found that a higher HAp concentration (40 wt%) effectively enhanced the mechanical properties (23.9 MPa), bioactivity, and protein adsorption. Cell experiments confirmed the non-toxicity and robust proliferation capability of the SA/HEC/HAp scaffold, likely attributed to the biocompatibility, bioactivity, strong cell adhesiveness, and excellent mechanical strength of HAp.^237^

The polymer scaffold is a critical component in nearly all tissue engineering approaches, aiming to replicate various functions of natural extracellular matrices.^238^ These matrices, composed of amino acids and sugar-based macromolecules, regulate tissue structure, cell function, and nutrient diffusion. Various polymers, such as aliphatic polyesters like poly(glycolic acid) (PGA), poly(lactic acid) (PLA), and copolymers (PLGA), have been extensively studied and utilized in tissue engineering.^239^ These polymers, considered safe in numerous medical contexts by the FDA, require incisions for the implantation of polymer/cell constructs. An intriguing approach for cell delivery in tissue engineering involves the use of injectable polymers, specifically hydrogels.^240^ This method allows clinicians to minimally invasively transplant cell–polymer combinations. Hydrogels, akin to the macromolecular components found in the body, are regarded as biocompatible and serve a variety of applications, including tissue engineering and drug delivery. Their recent application in tissue engineering includes functioning as scaffolds for the development of new tissues. Tissue engineering leverages innovative hydrogels based on cellulose technology, as highlighted by Radhakrishnan *et al.*,^241^ which act as frameworks resembling extracellular matrices to support the growth of new tissues. These frameworks create favourable conditions, providing essential space and nutrients crucial for targeted tissue development. Marler *et al.*,^242^ demonstrated their ability to engineer tissues such as cartilage, bone, muscle, skin, fat, arteries, ligaments, tendons, liver, bladder, and neurons. Furthermore, hydrogel frameworks contribute to surface modifications that facilitate tissue growth in biomedical implants. This modification can inhibit specific cell attachment while promoting the binding of others or securing biological components under specific conditions. Tallawi *et al.*,^243^ explained that these approaches control cellular interactions by reducing unwanted cell adhesion or improving connections between implanted biomaterials and bone or skin.

To address challenges associated with mesenchymal stem cell (MSC) culture, thermo-responsive hydrogels, particularly chitosan-*g*-PNIPAAm, present a promising solution. Lihui Peng *et al.*^244^ discussed the difficulties in MSC culture, especially the challenges in detaching MSCs from their culture carriers due to their strong adhesive properties, which often require harsh conditions. Therefore, further exploration of cell culture carrier materials and gentle methods for harvesting cultured cells is necessary. One potential strategy involves utilizing thermo-responsive hydrogels as cell culture carriers, which allow for easy detachment of cultured cells by simply lowering the temperature below their lower critical solution temperature (LCST). Chitosan-*g*-PNIPAAm demonstrates favourable characteristics for cellular attachment, proliferation, viability, and chondrocyte differentiation. This advancement led to the development of an injectable gel material based on chitosan/PNIPAAm for the chondrogenic differentiation of human MSCs. Subsequent assessments evaluated cartilage formation *in vivo* following the injection of a cell-thermo-sensitive gel complex. Animal experiments aimed to assess cartilage formation in the submucosal layer of rabbit bladders. Although the thermo-sensitive gel system may not be suitable for *in vivo* applications due to its high LCST of 32 °C, the innovative combination of chondrogenic ally differentiated MSCs with a thermo-sensitive polymer is proposed for use as an injectable cell–polymer complex. Observations of chondrogenic differentiation, both *in vitro* and *in vivo*, indicate the potential of this gel for a simplified treatment approach for vesicoureteral reflux using an endoscopic single injection technique, potentially eliminating the need for a dual injection system.

### 5.4. Smart materials

Cellulose-based hydrogels have emerged as crucial components in the development of advanced devices, particularly in the field of biochemical sensors. Their exceptional biocompatibility, substantial capacity for cell and small molecule storage, and low interfacial tension with aqueous solutions render them suitable for various applications.^184^ These sophisticated materials are essential for interpreting chemical information and translating specific analyte concentrations into comprehensive compositional analyses.

Typically, chemical sensors consist of two primary components: a chemical recognition system (receptor) and a physico-chemical transducer. Biosensors, a specialized subclass of chemical sensors, leverage biochemical mechanisms within their recognition systems.^183^ Recent studies have extensively explored the mechanical strength and biocompatibility of cellulose-based smart materials, which have applications ranging from electro-responsive electro-rheological (ER) suspensions to innovative composites with CNTs for conducting materials, wearable electronics, smart textiles, and various sensor types.^186–189^

Stimuli-responsive polymer systems are vital for effective transduction mechanisms, making hydrogels particularly well-suited for sensor applications.^245^ For instance, hydrogel films demonstrate exceptional sensitivity as pH-responsive nanosensors, characterized by rapid response times, highlighting their potential in diverse sensing applications.^190^ The rising demand for reliable, microfabricated chemical biosensors for real-time monitoring in biotechnology, food production, pharmaceuticals, and environmental sectors underscores the necessity for efficient and cost-effective analytical solutions. Functionalized hydrogel-coated biosensors can detect, transmit, and record variations in analyte concentration or the presence of specific functional groups, generating signals that correspond to the target analyte's concentration.^191^ The sensitivity of these hydrogels is influenced by various factors, including temperature, applied electrical voltage, pH, and the concentrations of organic compounds and salts in aqueous solutions. Stimuli-responsive hydrogels that can convert chemical energy into reversible mechanical work are becoming increasingly valuable in sensor technology. The swelling behaviour of pH-sensitive hydrogels, which is determined by the presence of acidic or basic groups within the polymer backbone, dynamically changes in response to pH shifts and charge interactions.^192,193^

The development of smart wearable devices has become a prominent research focus due to their potential in health monitoring.^246^ Self-healing wearable devices can restore their structure and functionality after damage, enhancing durability and safety.^247^ Self-healing hydrogels, as soft and flexible materials, are particularly appealing for the development of wearable pressure sensors that detect human motion.^248^ To meet the required mechanical toughness and mobility properties, two seemingly contradictory characteristics, self-healing hydrogels must effectively balance dynamic cross-links for healing and stable cross-links for mechanical strength. The integration of self-healing abilities with robust mechanical properties into a single conductive hydrogel presents a significant challenge. CNCs are commonly utilized as fillers to reinforce hydrogels due to their unique properties, including mechanical strength, biodegradability, biocompatibility, and modifiability. Recent advancements have successfully employed CNCs to create conductive hydrogels that exhibit both self-healing and mechanical performance.^29,30^

Inspired by the hierarchical structures of biological soft tissues, Liu *et al.* developed a conductive, elastic, self-healing, and strain-sensitive functional network hydrogel (F-hydrogel) formed from a “soft” homogeneous polymer network *via* covalent cross-linking of polyvinyl alcohol (PVA) and polyvinylpyrrolidone (PVP).^249^ This network incorporated a “hard” Fe^3+^-cross-linked CNC network with dynamic CNC-Fe^3+^ coordination bonds as reinforcing domains.^250^ Under stress, these dynamic coordination bonds functioned as sacrificial bonds to efficiently dissipate energy, while the PVA-PVP network facilitated smooth stress transfer. As a result, the F-hydrogels exhibited exceptional mechanical properties, characterized by a tensile strength of 2.1 MPa, toughness of approximately 9.0 MJ m^−3^, and stretchability of 830%.^249^ These properties allow the hydrogels to withstand significant deformations, such as compression and knotting, without visible damage. Furthermore, the F-hydrogels demonstrated autonomous self-healing capabilities within just 5 minutes, without external stimuli or healing agents, while maintaining their sensing performance before and after restoration. They exhibited ultrasensitive, stable, and repeatable resistance variations in response to mechanical deformations, making them promising candidates for wearable devices.

**Fig. 14:**
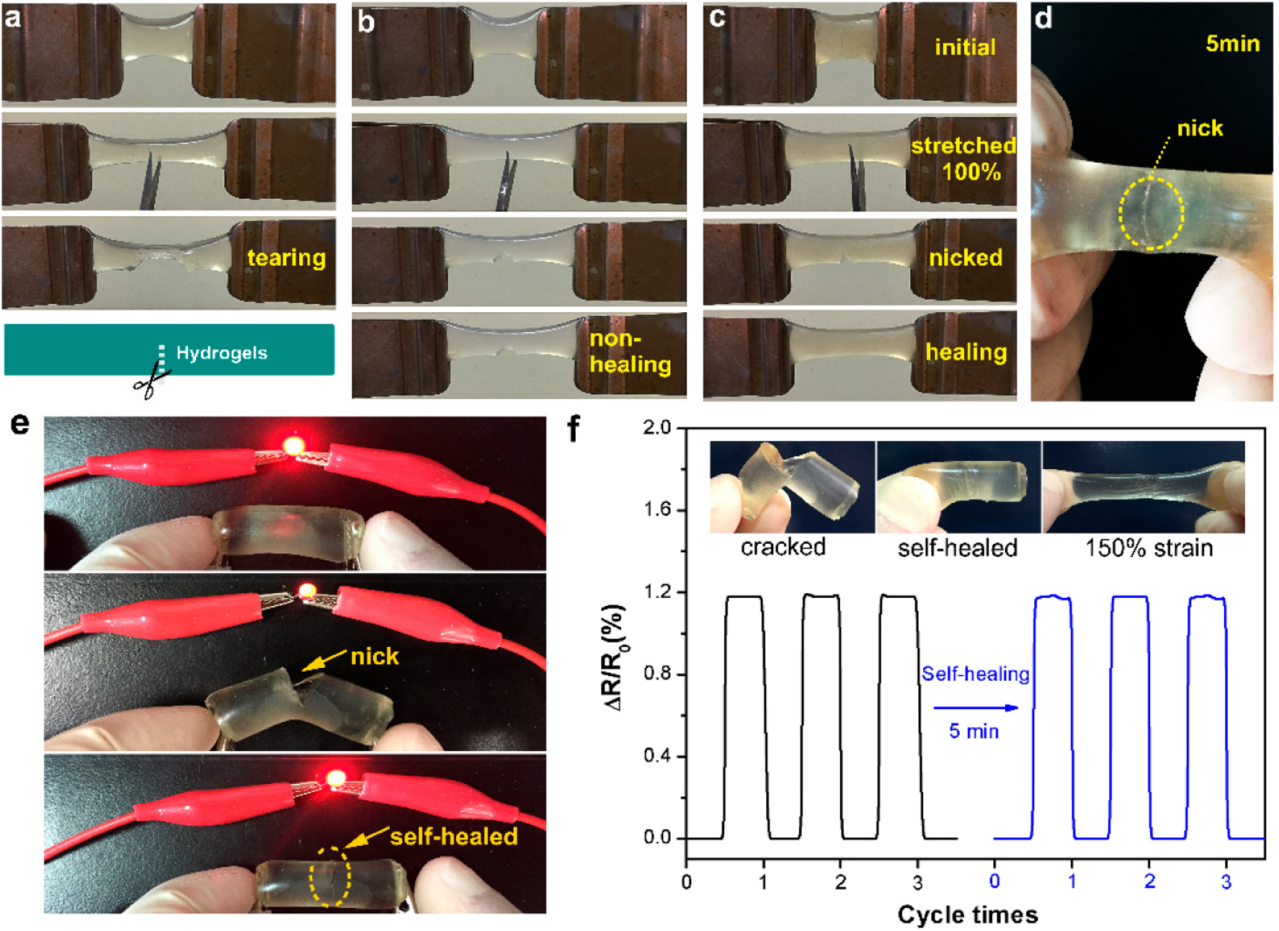
Real images of notch sensitivity testing. This series of photographs illustrates the notch sensitivity experiments conducted on hydrogels. A notch (one-third of the width) was created in the edge of the hydrogels, which were initially stretched to 100% using scissors: (a) the notch in the tearing H-hydrogels, (b) the undamaged notch in C-hydrogels-15, and (c) the intact notch demonstrating self-healing in F-hydrogels-15/2.5. (d) Evidence of self-healing in F-hydrogels-15/2.5 is shown after only 5 minutes post-unloading, occurring without any external stimuli or healing agents. (e) Changes in the LED light within the electric circuit are depicted before and after the self-healing process. (f) The sensor performance of the F-hydrogels during a loading–unloading cycle at 150% strain is shown, along with an inset featuring photographs of the F-hydrogels in cracked, self-healed, and 150% strain conditions. This image is adopted from the ref. 249. Copyright © 2017 American Chemical Society.

Based on the characteristics of the F-hydrogels, a wearable soft strain sensor was assembled by Liu *et al.*,^249^ to monitor finger joint motions, breathing patterns, and slight blood pulses (Fig. 14). As the bending angle of the finger increased from 0° to 120° during a controlled bending process, the relative resistance of the F-hydrogel sensor increased correspondingly (Fig. 14(a)). The sensor displayed repeatable responses at a bending angle of 90° with a working frequency of 0.3 Hz (Fig. 14(a)). When attached to the skin of a volunteer's lower left rib cage, the sensor effectively monitored and distinguished various breathing modes-including regular breathing, rapid deep breathing, and breath-holding-based on changes in relative resistance (Fig. 14(b)). Additionally, when placed on the wrist of a volunteer (Fig. 14(c)), the sensor accurately reflected changes in blood pulse before and after exercise, as shown on the relative resistance curve (Fig. 14(d)), confirming its high strain sensitivity and rapid response-ideal for human health monitoring.^249^

Similarly, Shao *et al.*,^251^ developed a tough, self-healing, and self-adhesive gel by constructing synergistic multiple coordination bonds among tannic acid (TA)-coated CNCs, PAA chains, and Al^3+^ ions within a covalent polymer network. The TA@CNCs acted as dynamic bridges in a hierarchically porous network, mediated by multiple reversible coordination bonds. This architecture endowed the PAA-TA@CNCs-Al^3+^ gels with superior mechanical properties, including ultra stretchability (fracture strain of 2952%), high compression performance (95% strain without fracture), and toughness (5.60 MJ m^−3^). The dynamic features of the reversible coordination bonds also provided the gels with excellent recoverability and electrical self-healing properties. Incisions in the gels self-healed automatically, disappearing nearly completely within 30 minutes, achieving an equilibrium state with a healing efficiency of 92%. Additionally, due to the presence of catechol groups from the incorporated TA, these gels displayed durable and repeatable adhesion to various substrates, including human skin, rubber, aluminium, glass, and polytetrafluoroethylene, without inducing inflammatory responses or leaving residues. This makes them suitable as flexible strain sensors directly adhered to the skin.

Owing to their excellent self-adhesiveness, high strain sensitivity, remarkable electrical stability, and fast self-healing capabilities, these gels can detect substantial motions, such as joint bending and stretching during various human activities, including opisthenar, elbow, and shoulder joint bending in standard shooting actions.^251^ Furthermore, subtle motions like pulse and breath, crucial for real-time healthcare monitoring during sports training, can be accurately detected and promptly recognized by the gel strain sensors.^252^ This research paves the way for developing biocompatible cellulose-based conductive hydrogels for applications in wearable electronic sensors, healthcare monitoring, and even soft intelligent robots.

Pressure sensors have become indispensable in the realm of wearable devices due to their outstanding sensing capabilities.^253^ These sensors offer a wide range of applications, including pulse detection, voice recognition, and motion sensing. A notable example is the work by Li *et al.*,^254^ who utilized a simple pyrolysis technique to produce carbon cottons (CCs) with remarkable properties such as low density and an electrical conductivity of approximately 11 S m^−1^, utilizing a cotton substrate. The composite material was synthesized by infusing polydimethylsiloxane (PDMS) resin into the CC structure under vacuum conditions. The resulting CC/PDMS composite pressure sensor exhibited a high sensitivity of 6.04 kPa^−1^, a large operational pressure ranges up to 700 kPa, a broad frequency response from 0.01 Hz to 5 Hz, and outstanding durability, maintaining performance over 1000 cycles. The practical application of this sensor was demonstrated by embedding it in a sports shoe and a waist belt, where it was used to monitor health metrics and track athletic performance. The sensor's manufacturing process is cost-effective and scalable, primarily due to the use of cotton, a readily available and inexpensive material. These attributes make this sensor highly promising for future applications in health monitoring and the development of wearable electronics, such as prosthetic skins.

**Fig. 15:**
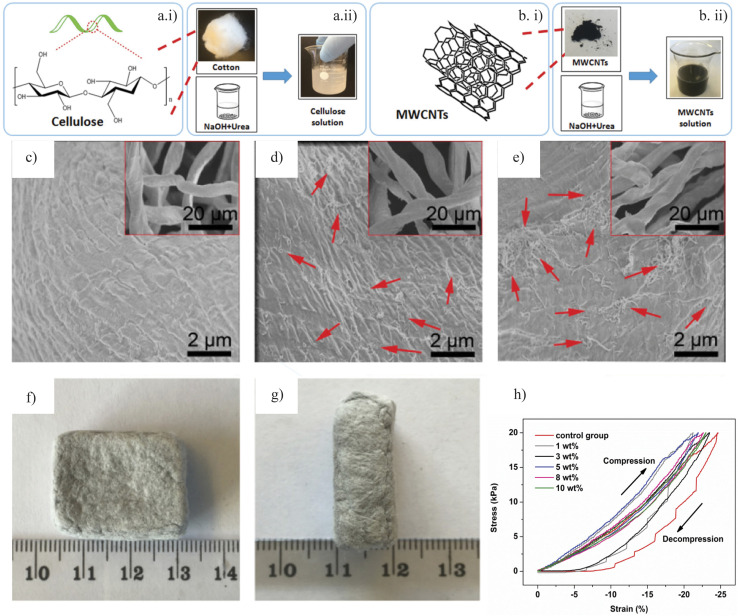
(a, i, and ii) Depicts the chemical structure of cotton cellulose and illustrates its swelling behaviour. (b, i, and ii) Shows the chemical structure of multi-walled carbon nanotubes (MWCNTs) and their dispersion characteristics. (c) Presents a SEM image of the pristine cellulose network in its substrate form. (d and e) Demonstrates pressure sensors incorporating varying mass fractions of MWCNTs, with concentrations of (d) 1 wt% and (e) 3 wt%. (f) Provides a planar view of the strain sensor, while (g) displays its axial view. (h) Depicts the stress–strain curves for the pressure sensor, as shown in the referenced work. Reproduced with permission from ref. 255. Copyright © 2019 American Chemical Society.

In a different approach, cellulose fibres were coated with multi-walled carbon nanotubes (MWCNTs) to create flexible and pressure-responsive sensors.^255^ The team employed cotton cellulose in combination with MWCNTs to fabricate these sensors and analysed their morphology using SEM (Fig. 15).^255^ The mechanical performance of the sensors, in terms of stress–strain behaviour, was assessed for varying MWCNT compositions. These sensors exhibited key advantages such as flexibility, porosity, cost-effectiveness, and significant potential as textiles for various applications. Further innovation was seen in the work of Li *et al.*,^256^ who developed conductive fibres to fabricate a highly stable and sensitive textile sensor with excellent electrical properties. In this process, silver nanocomposite particles and elastic rubber were combined to fabricate the conductive fibres *via* a coating method, achieving an electrical conductivity of 0.15 Ω cm^−1^. These fibres formed a well-organized electrical network, demonstrating both durability and resistance to external forces for about 3000 bending cycles. The inclusion of elastic rubber enhanced the fibres' stretchability. Additionally, the researchers employed PDMS-coated conductive fibres to build a textile-based capacitive pressure sensor. This sensor exhibited remarkable features, including ultra-high sensitivity of around 0.21 kPa^−1^ in the low-pressure region, a rapid response time of under 10 ms, exceptional durability for over 10 000 cycles, and minimal hysteresis. The textile sensor was then woven into a fabric, effectively creating a skin-like interface for human–machine interactions. With the potential to be seamlessly integrated into clothing and gloves, these sensors offer exciting possibilities for wireless human motion tracking, making them strong candidates for smart textile applications.

Capacitive sensors, typically composed of two parallel plates separated by a dielectric material such as air, are well-known for their superior linearity and minimal hysteresis, characteristics that make them particularly effective for a wide range of practical applications. In recent research, integrating single-walled carbon nanotube (SWCNT) paper into a PDMS matrix has enabled the development of flexible, highly sensitive strain sensors. For example, Zhou *et al.*,^257^ demonstrated a novel approach where SWCNT paper-based sensors achieved an impressive gauge factor (GF) of 107 when subjected to a 50% applied strain. This fabrication technique allows for the incorporation of SWCNT paper of varying thicknesses within PDMS substrates, resulting in a range of smart materials with distinct properties, such as enhanced sensitivity and moderate flexibility. To optimize the performance of these sensors, the introduction of inter- or intra-laminar cracks has proven effective, with each method influencing the sensor's response in unique ways. This innovative strategy not only holds promise for strain detection but also presents an opportunity for expanding the use of other cellulose derivatives or nanomaterials, paving the way for the development of highly effective strain sensors.^257^

In another significant breakthrough, researchers have designed a flexible, ultra-sensitive pressure sensor utilizing a simple, cost-effective, and scalable approach based on silver nanoparticle (AgNP)-poly(3,4-ethylenedioxythiophene) (PEDOT)-paper composites.^258^ This sensor consists of intertwined fibres coated with a conductive PEDOT layer, supported by a permeable and stretchable paper substrate. The PEDOT coating enhances the electrical conductivity of the cellulose fibres, while the addition of AgNPs to the PEDOT matrix increases the surface roughness of the polymer, significantly improving its conductive properties. These modifications-lowered resistance and increased surface roughness-lead to notable improvements in sensor performance, particularly in terms of sensitivity and detection range. The sensor demonstrates an impressive sensitivity of 0.119 kPa^−1^ within a pressure range of 0–12 kPa, along with excellent stability over 2000 cycles. These outstanding characteristics make the sensor highly suitable for applications in human movement detection, including monitoring of breathing, phonation, pulse, heartbeat, and voice recognition. Furthermore, the sensor has been successfully integrated into a human–machine interaction system, enabling voice recognition and even musical translation from a piano keyboard. This advancement not only creates new opportunities for bi-directional communication channels but also facilitates the integration of real-time human behaviour monitoring, advanced digital signal processing, and artificial intelligence technologies.^258^

## 6. Conclusions and future aspects

Cellulose-based hydrogels hold immense promise for biomedical applications due to their biocompatibility, biodegradability, and versatile mechanical properties.^259,260^ As we look ahead, further advancements in cellulose-based hydrogel technology will open up new avenues for their use in regenerative medicine, drug delivery, wound healing, and tissue engineering. One key area of focus will be enhancing the mechanical toughness and swelling behaviour of these hydrogels simultaneously to meet the demanding requirements of biomedical applications.^1,261,262^

By incorporating dynamic noncovalent interactions, the hydrogel can exhibit reversible responses to external stimuli, enabling dynamic changes in structure and properties.^263,264^ This adaptability allows the hydrogel to withstand mechanical stresses and undergo controlled swelling without compromising its integrity. Simultaneously, the introduction of non-reversible covalent bonds provides stability and permanence to the hydrogel network, ensuring long-term structural integrity and resistance to degradation. These covalent bonds act as anchors within the hydrogel matrix, reinforcing its mechanical strength and preventing excessive swelling.

**Fig. 16:**
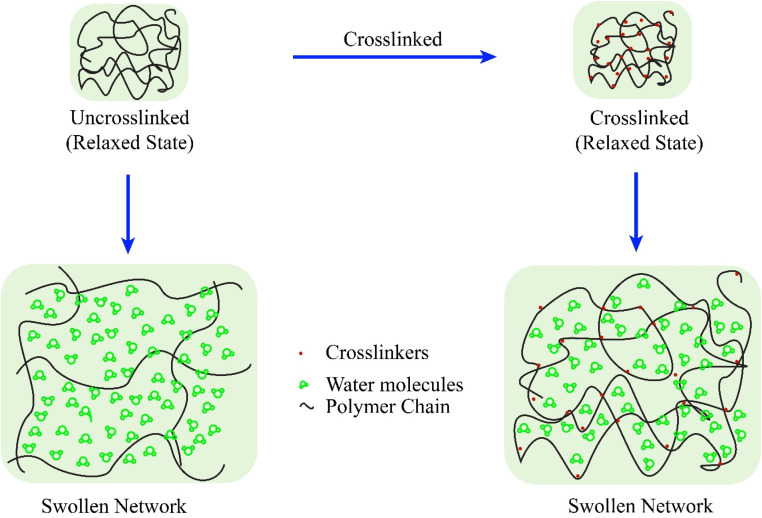
Swollen network structure of uncross-linked and cross-linked hydrogels.

The synergistic combination of dynamic noncovalent interactions, such as HB and host–guest interactions, with non-reversible covalent bonds offers a promising approach to overcome the limitations associated with mechanical toughness and swelling behaviour in cellulose-based hydrogels. Through the synergistic interplay between dynamic noncovalent and non-reversible covalent bonds, cellulose-based hydrogels can achieve a balance between toughness and swelling behaviour, making them ideal candidates for biomedical applications. The dynamic noncovalent interactions allow for reversible deformation and toughening, while the non-reversible covalent interactions provide structural stability and control over swelling behaviour. This synergistic approach can lead to the development of cellulose-based hydrogels with enhanced properties for biomedical applications, paving the way for new advances in regenerative medicine, drug delivery, and tissue engineering. Here Fig. 16 represent the network structure of crosslinked and uncross linked hydrogels at swollen condition. Herein, the uncross linked gel network absorbed a larger amount of water in an uncontrolled manner compared to the crosslinked network. However, after certain time, the uncross linked hydrogels get disintegrated while the crosslinked hydrogel remained same. However, the proper combination of those techniques would be offering a combination of mechanical toughness and control swelling properties.

There is limited understanding regarding the establishment of theoretical models that can quantitatively predict the mechanical properties of cellulose-based hydrogels based on their composition. Factors such as sacrificed physical and chemical bonds, degree of homogeneity, cross-linking density, concentration, entanglement, and network structure all play crucial roles.

Molecular simulations are essential for advancing the study of cellulose-based hydrogels. These simulations can (i) expedite the modelling of large and complex hydrogel structures; (ii) develop precise force fields to analyse the contributions of various factors; (iii) assess the mechanical properties of specific compositions before experimental testing; and (iv) design cellulose-based hydrogels tailored to specific applications, such as ion and electron transport in energy storage and conversion. Additionally, it is important to elucidate the mechanical behaviour of these hydrogels around their yield point.

Cellulose-based smart materials offer immense potential for advancing wearable sensors, yet significant challenges persist in improving their biocompatibility, multi-functionality, and seamless integration with human health monitoring systems. Future research should prioritize the creation of innovative microstructures, the development of skin-like electronics with optimized mechanical and electrical properties, and the application of advanced processing techniques to produce intelligent, multi-functional devices. Furthermore, overcoming limitations in sensitivity, stretchability, and wireless functionality is crucial for transitioning these materials into commercially viable, scalable, and durable technologies. Such advancements promise not only to revolutionize health monitoring but also to expand their utility into areas like drug delivery and broader biological research, heralding groundbreaking progress in biomedical engineering.

Research on cellulose-based hydrogels for various innovative applications is still in its early stages. Potential applications include injectability and 3D printing, optical, electric, and magnetic tough materials, actuators, ionotropic devices, flexible electronic skins, and impact-resistant materials. Despite being in the nascent phase, the inherent mechanical properties and cytocompatibility of cellulose-based hydrogels offer promising pathways for their use across multiple fields.

## Abbreviations

CMC: Carboxymethyl cellulose
EC: Ethyl cellulose
MC: Methylcellulose
HEMC: Hydroxyethyl methyl cellulose
MCC: Microcrystalline cellulose
HBI: Hydrogen bonding interactions
EIS: Electrostatic interactions
PAA: Polyacrylic acid
HPC: Hydroxypropyl cellulose
CNCs: Cellulose nanocrystals
CC: Chemical crosslinked
Upy: 2-Ureido-4-pyrimidone
PC: Physical crosslinked
CNFs: Cellulose nanofibrils
CSH: Cellulose/silk fibroin hydrogel
DTT: 1,4-Dithiol-dl-threitol
CA: Citric acid
CYS: Cysteamine dihydrochloride
SD: Swelling degree
SR: Swelling ratio
ES: Equilibrium-swelling theory
RE: Rubber-elasticity
PGA: Poly(glycolic acid)
PLGA: Copolymers
IPN: Interpenetrating network
PLA: Poly(lactic acid)
HEC: Hydroxyethyl cellulose
SA: Sodium alginate
DVS: Divinyl sulfone
PNIPAAm: (*N*-isopropylacrylamide)
MSC: Mesenchymal stem cell
PDMS: Polydimethylsiloxane
CYS: Cysteamine dihydrochloride
AG: Alginate
ECH: Epichlorohydrin
OP: Osmotic pressure

## Data availability

There is no supporting document.

## Author contributions

Conceptualization: M. M. H. R.; original manuscript writing, M. M. H. R., figure drawing: M. M. H. R.; revision and editing, M. M. H. R., The author has read and agreed to the published version of the manuscript.

## Conflicts of interest

The author declares no conflict of interest.
